# Body and peripersonal space representations in chronic stroke patients with upper limb motor deficits

**DOI:** 10.1093/braincomms/fcac179

**Published:** 2022-08-05

**Authors:** Michela Bassolino, Matteo Franza, Eleonora Guanziroli, Giuliana Sorrentino, Elisa Canzoneri, Maria Colombo, Andrea Crema, Tommaso Bertoni, Giulio Mastria, Matteo Vissani, Arseny A Sokolov, Silvestro Micera, Franco Molteni, Olaf Blanke, Andrea Serino

**Affiliations:** Laboratory of Cognitive Neuroscience, Center for Neuroprosthetics and Brain Mind Institute, School of Life Science, Swiss Federal Institute of Technology (EPFL), Geneva 1202, Switzerland; Department of Clinical Neuroscience, Centre Hospitalier Universitaire Vaudois (CHUV), MySpace Lab, Lausanne 1011, Switzerland; Institute of Health, School of Health Sciences, HES-SO Valais-Wallis, Sion 1950, Switzerland; Laboratory of Cognitive Neuroscience, Center for Neuroprosthetics and Brain Mind Institute, School of Life Science, Swiss Federal Institute of Technology (EPFL), Geneva 1202, Switzerland; Villa Beretta Rehabilitation Center, Valduce Hospital Como, Costa Masnaga 23845, Italy; Laboratory of Cognitive Neuroscience, Center for Neuroprosthetics and Brain Mind Institute, School of Life Science, Swiss Federal Institute of Technology (EPFL), Geneva 1202, Switzerland; Laboratory of Cognitive Neuroscience, Center for Neuroprosthetics and Brain Mind Institute, School of Life Science, Swiss Federal Institute of Technology (EPFL), Geneva 1202, Switzerland; Villa Beretta Rehabilitation Center, Valduce Hospital Como, Costa Masnaga 23845, Italy; Bertarelli Foundation Chair in Translational Neuroengineering, Centre for Neuroprosthetics and Institute of Bioengineering, School of Engineering, Swiss Federal Institute of Technology (EPFL), Geneva 1202, Switzerland; AGO Neurotechnologies, Sàrl, Geneva 1201, Switzerland; Department of Clinical Neuroscience, Centre Hospitalier Universitaire Vaudois (CHUV), MySpace Lab, Lausanne 1011, Switzerland; Department of Clinical Neuroscience, Centre Hospitalier Universitaire Vaudois (CHUV), MySpace Lab, Lausanne 1011, Switzerland; Laboratory of Cognitive Neuroscience, Center for Neuroprosthetics and Brain Mind Institute, School of Life Science, Swiss Federal Institute of Technology (EPFL), Geneva 1202, Switzerland; The Biorobotics Institute and Department of Excellence in Robotics and AI, Scuola Superiore Sant’Anna, Pontedera, Pisa 56025, Italy; The Wellcome Trust Centre for Neuroimaging, Institute of Neurology, University College London, London WC1N 3BG, UK; Service de Neurologie, Département des Neurosciences Cliniques, Centre Hospitalier Universitaire Vaudois (CHUV), Lausanne 1011, Switzerland; Bertarelli Foundation Chair in Translational Neuroengineering, Centre for Neuroprosthetics and Institute of Bioengineering, School of Engineering, Swiss Federal Institute of Technology (EPFL), Geneva 1202, Switzerland; The Biorobotics Institute and Department of Excellence in Robotics and AI, Scuola Superiore Sant’Anna, Pontedera, Pisa 56025, Italy; Villa Beretta Rehabilitation Center, Valduce Hospital Como, Costa Masnaga 23845, Italy; Laboratory of Cognitive Neuroscience, Center for Neuroprosthetics and Brain Mind Institute, School of Life Science, Swiss Federal Institute of Technology (EPFL), Geneva 1202, Switzerland; Department of Clinical Neuroscience, University of Geneva Medical School, Geneva 1211, Switzerland; Laboratory of Cognitive Neuroscience, Center for Neuroprosthetics and Brain Mind Institute, School of Life Science, Swiss Federal Institute of Technology (EPFL), Geneva 1202, Switzerland; Department of Clinical Neuroscience, Centre Hospitalier Universitaire Vaudois (CHUV), MySpace Lab, Lausanne 1011, Switzerland

**Keywords:** stroke, motor deficits, body representations, peripersonal space, lesion analysis

## Abstract

The continuous stream of multisensory information between the brain and the body during body–environment interactions is crucial to maintain the updated representation of the perceived dimensions of body parts (metric body representation) and the space around the body (the peripersonal space). Such flow of multisensory signals is often limited by upper limb sensorimotor deficits after stroke. This would suggest the presence of systematic distortions of metric body representation and peripersonal space in chronic patients with persistent sensorimotor deficits. We assessed metric body representation and peripersonal space representation in 60 chronic stroke patients with unilateral upper limb motor deficits, in comparison with age-matched healthy controls. We also administered a questionnaire capturing explicit feelings towards the affected limb. These novel measures were analysed with respect to patients’ clinical profiles and brain lesions to investigate the neural and functional origin of putative deficits. Stroke patients showed distortions in metric body representation of the affected limb, characterized by an underestimation of the arm length and an alteration of the arm global shape. A descriptive lesion analysis (subtraction analysis) suggests that these distortions may be more frequently associated with lesions involving the superior corona radiata and the superior frontal gyrus. Peripersonal space representation was also altered, with reduced multisensory facilitation for stimuli presented around the affected limb. These deficits were more common in patients reporting pain during motion. Explorative lesion analyses (subtraction analysis, disconnection maps) suggest that the peripersonal space distortions would be more frequently associated with lesions involving the parietal operculum and white matter frontoparietal connections. Moreover, patients reported altered feelings towards the affected limb, which were associated with right brain damage, proprioceptive deficits and a lower cognitive profile. These results reveal implicit and explicit distortions involving metric body representation, peripersonal space representation and the perception of the affected limb in chronic stroke patients. These findings might have important clinical implications for the longitudinal monitoring and the treatments of often-neglected deficits in body perception and representation.

## Teaser

Bassolino *et al*. studied body perception in post-stroke chronic patients with upper limb sensorimotor deficits. Results reveal distortions in the perceived dimensions, representation of peripersonal space and explicit feelings towards the affected limb. This motivates quantitative assessments of body perception even in later phases after stroke.

## Graphical Abstract

**Graphical Abstract:**
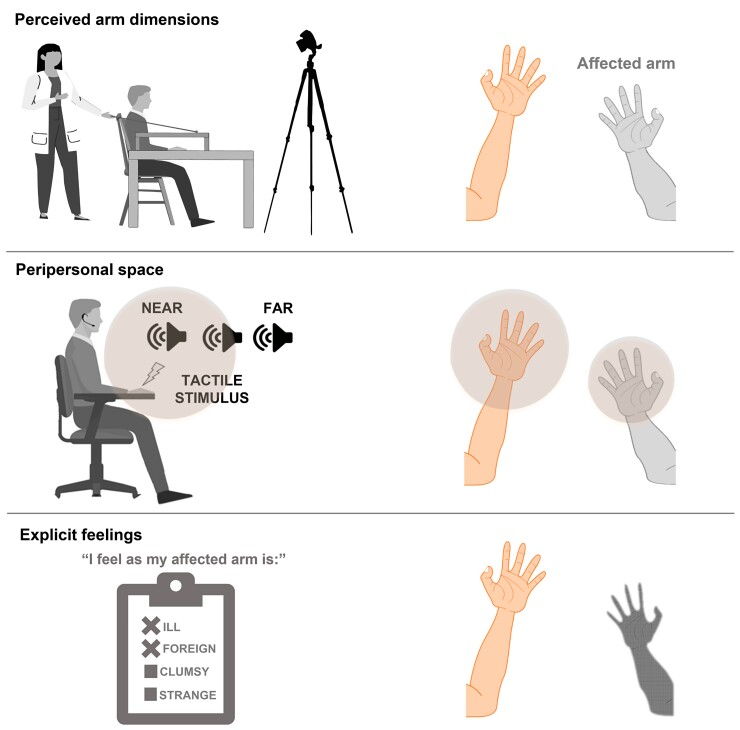

## Introduction

Post-stroke patients frequently present upper limb motor and sensory impairments.^1–5^ In addition, severe alterations in how patients experience their affected body parts, such as asomatognosia, somatoparaphrenia,^6–11^ anosognosia for hemiplegia^12–14^ or personal neglect,^15^ are mainly seen in the acute phase in post-stroke patients, especially if involving right brain damage (RBD).

However, distortions in body perception, and the underlying body representations (BR), are not only limited to those disorders, do not involve only RBD patients and can extend to chronic patients. For instance, disorders such as apraxia, autotopagnosia, and body-specific aphasia, interpreted as disturbance of specific BR (respectively, body schema, body structural description and body semantics), have been reported both in RBD and LBD post-stroke patients.^16–18^ Moreover, an aberrant form of embodiment towards another person’s limb, when this limb is located in a position congruent with the location of the patient’s contralesional side, called pathological embodiment, has been described both in RBD and LBD patients, also in the chronic phase.^19–22^ Similarly, long-lasting cases of heterotopagnosia^23,24^ or feelings of disownership towards the affected limb have also been reported in chronic patients.^8^ These disturbances in BR could negatively affect the recovery.^25–27^

Though, so far, rare experimental studies have been conducted in post-stroke patients^16,17,28^ with chronic motor impairments,^29^ by using rigorous experimental measures, to assess alterations in BR. In particular, studies in stroke patients with motor deficits have rarely assessed representations that are considered crucial for body–environment interactions, such as the metric representation of the body^19,29^ (mBR) capturing the perceived dimension of the body parts,^30–34^ and the representation of the space around the body, i.e. peripersonal space (PPS). Specifically, the mBR is considered crucial for action,^30,35,36^ by providing essential dimensional cues of the body parts involved in the movement, such as the length and the width of the hand and arm during a grasping movement towards a target. Such metric information is not provided by a single sensory system, but by multiple body signals (visual, proprioceptive, tactile cues) that build and update the mBR.^30,36,37^ Moreover, multisensory and motor bodily signals contribute to represent the space around the body, the PPS, where physical interactions between one’s own body and external stimuli occur.^38–40^ PPS is coded by a special mechanism of multisensory interaction, whereby tactile processing of bodily stimuli is affected by external (visual or acoustic) stimuli presented close, but not far, from the body in body part-centred reference frames.^41–43^ PPS representation seems to support both active and defensive interactions in the environment, and underlies a self-other distinction, involved in self-consciousness.^38,39^

Importantly, both mBR and PPS are plastically updated by sensorimotor experiences, as shown after tool use,^31,38,44–49^, limb disuse^50^ or in case of changes in the physical structure of the body, such as amputation.^51,52^

Thus, given that mBR and PPS depend on sensorimotor information, and that sensorimotor functions can be impaired after stroke, stroke patients with sensorimotor deficits may present important and persistent alterations in mBR and PPS. Testing this hypothesis could have important clinical implications to improve assessment and in turn design more tailored rehabilitative approaches.^53^

With this goal, here we applied well-known tasks assessing the perceived dimensions and shape of the upper limb (body-landmarks localization task, BLT)^32,35,50,54^ and the multisensory representation of the PPS (audio-tactile interaction task, hereafter PPS task)^32,41,42,50^ in a sample of 60 chronic stroke patients with persistent motor deficits in comparison with age-matched healthy controls. Additionally, we adapted a questionnaire to assess whether explicit disturbances in the affected upper limb perception, usually reported in acute patients, are present in the chronic phase (affected limb explicit feeling questionnaire, ALEFq, see Crema *et al*.^53^).

## Materials and methods

### Participants

Sixty chronic stroke patients with unilateral, upper limb motor deficits were included in the study (see Supplementary Table 1). They also took part in an interventional clinical trial (ClinicalTrials.gov identifier: NCT03349138) testing the efficacy of a new neuromuscular electrical stimulation for upper limb rehabilitation.^53^ Data included in the present work regard the extensive baseline evaluation performed before any treatment. The major inclusion criteria were age >18 years, one or more strokes at least 6 months before the study enrolment, unilateral functional impairments of the contralesional upper limb (Motricity Index < 85) and right-handedness. Besides specific criteria related to the interventions, listed in Crema *et al*.,^53^ the main exclusion criteria were pain due to spasticity (Modified Ashworth Scale >2), previous major neurological or psychiatric disorders and cognitive deficits preventing comprehension of instructions to perform the proposed tasks. Patients were evaluated at Villa Beretta Rehabilitation Center, Costa Masnaga, Lecco, Italy. Detailed clinical characteristics of all patients are provided in Supplementary Tables 1 and 2.

Patients were compared with age-matched, right-handed, healthy controls whose data were partially reported previously in another study.^32^ All controls had a normal or corrected-to-normal vision, hearing and touch, no psychiatric nor neurological deficits, no cognitive impairments (Montreal Cognitive Assessment: all equivalent scores ≥ 2), no pain, no fractures (in the previous 12 months) nor sensorimotor deficits in the upper limbs.

All participants were naïve to the purpose of the study and participated after giving informed consent. The study was conducted with the approval of the local ethics committees (Commission Cantonale Valaisanne d'Ethique Médicale, CCVEM 017/14 and CE Interaziendale delle Province di Lecco Como e Sondrio, 48/2016). The manuscript was prepared according to the STROBE checklist for case–control studies. Further information and a CONSORT flow diagram for the inclusion of patients can be found in Crema *et al*.^53^

### Patients’ assessments

#### Experimental testing

We used the BLT to assess the implicit perceived dimension of the upper limbs and a PPS task to evaluate the multisensory features of PPS. These tasks are described elsewhere^32,53^ and summarized below (see Supplementary material for further details). The ALEFq,^53^ which captures explicit disturbances in the affected limb perception (see Supplementary Table 3), as well as an additional task targeting the explicit perceived dimension of the upper limbs (avatar adjustment task, AAT)^32^ are described in Supplementary material.

#### Implicit body representation task: the BLT

To assess the implicit perceived dimension of the upper limbs, we adapted the BLT.^32,33,36,49,50,54,55^ Participants had to verbally indicate when a moving marker reached the felt position of one of five possible non-visible anatomical landmarks that were: the tip of the index finger, the tip of the annular finger, the internal part of the wrist (the radius styloid), the external part of the wrist (the ulnar styloid) and the elbow joint (the olecranon) (see Supplementary material for further details and the Graphical abstract for a figure of the task). The width or length of the body parts was then computed *a posteriori* during the data analysis.^56^

For each participant, we calculated an index of the bias in the perceived dimension with respect to the actual one (*estimated dimension*)^32,35^ as the ratio between the perceived and the real size (length and width) of the arm and the hand (four parameters). Values below or above 1 represent, respectively, an underestimation or overestimation of the perceived dimension with respect to the real one. Moreover, similar to previous studies,^32^ we calculated a global index of the perceived shape of the arm and the hand, as the ratio between the estimated dimension on the width and the length, for both arm and hand (*normalized shape index*). In this case, a value higher than 1 indicates a higher estimated dimension for the width with respect to the length.

#### PPS task

Participants were asked to answer as fast as possible to the tactile stimulation on the hand by pressing a foot pedal (with their right foot for controls, and with the unaffected foot for patients) and to ignore the non-informative looming sound (see Supplementary material for details and the Graphical abstract for a figure of the task). The crucial manipulation of this task consists of the fact that the tactile stimulus was randomly presented at one out of three temporal delays (D3, D2 and D1) from the sound onset, i.e. when the sound was perceived at one out of three possible distances from the body (*D*3/far = 0.3 s; *D*2/medium = 1.5 s; *D*1/near = 2.7 s). The correspondence between the temporal interval from the sound onset and the spatial distance between the sound and the touch location matched linearly and negatively. Similarly, also unimodal trials (only tactile) were administered at three different delays, corresponding to the equivalent timing of the farthest, medium and nearest distance of sound. In line with the literature,^41,42^ we expect that, when the acoustic stimulus is located within the boundaries of the hand-PPS, sounds interact in a multisensory way with tactile processing, resulting in faster reaction times (RTs) to tactile stimuli delivered to the hand, compared with unimodal tactile stimulation.

We calculated the mean *RTs to audio-tactile stimuli* for every temporal delay and we removed from the analysis all the trials exceeding 2 standard deviations from the mean RT (outlier trials). Preliminary analyses on unimodal RTs were conducted in the two groups (see Supplementary material for the results). According to previous works,^32,38,41,57^ unimodal trials were considered as a baseline and the *audio-tactile RTs* of each participant were corrected by subtracting the baseline unimodal condition, that is the fastest mean RT among the unimodal tactile conditions. In this way, negative values indicate a multisensory facilitation effect on tactile RTs due to auditory stimulation with respect to the fastest unimodal tactile RT.^32,38,41,57^ The baseline-corrected audio-tactile RTs were compared between patients and controls.

#### Clinical scales and lesions

Patients’ motor, sensory and cognitive abilities were evaluated at the time of experimental testing using standard clinical scales. Neuroradiological images (either magnetic resonance imaging or computerized tomography) performed for clinical purposes in the acute or chronic phase were used for lesion analyses. All details are in Supplementary material.

### Statistical analyses

#### Main analyses: linear mixed models

##### General aspects

To test differences between patients and controls, data from the experimental tasks (BLT: estimated dimensions and normalized shape index; PPS: audio-tactile RTs) were compared by using a linear mixed model (LMM), similar to a previous study.^32^ The use of LMM was supported by a model selection based on Akaike’s information criterion (AIC) and Bayesian information criterion (BIC), revealing that LMM is always better than ANOVA. In all the analyses, participants were considered as a random effect, together with additional random effects added on the basis of a model selection with AIC and BIC criteria. For fixed effects, *P*-values were obtained by likelihood ratio tests, and the degree of freedom was approximated using the Satterthwaite method. Because of previously showed age-related effects in body and space representations evaluated through the BLT and PPS tasks,^32^ the age of participants was inserted as a covariate in all models. To explore significant interactions, when necessary, follow-up analyses or the Tukey *post hoc* test was used. All the analyses were conducted using LMM with the software R (R Core Team, 2017, http://www.R-project.org/).

The analyses were conducted with the same following approach for both tasks.

##### Body-landmarks localization tasks

For both the estimated dimension and the normalized shape index, we first considered only data collected in patients to test if a main effect or interaction of patients’ brain lesion: brain ‘lesion lateralization’ (two levels: RBD, LBD), ‘limb’ (affected, unaffected) and ‘upper limb dimensions’ (arm length, arm width, hand length, hand width) were used as fixed factors. If a main effect or interaction of brain lesion emerged, RBD and LBD patients were considered separately for further comparisons with the control group; otherwise, all patients were considered together. Then, to compare data obtained in patients and control participants, we used the factor ‘limb’ which levels were coded as nested (4 levels: affected/unaffected, belonging to the patients and left/right belonging to the controls) and ‘upper limb dimensions’ as the fixed factor. This allows us to directly (i) compare possible asymmetry between the two sides in patients and controls,^32^ (ii) compare each side of controls and patients, without imposing planned comparisons and with appropriate corrections and (iii) limit the numbers of nested factors and the complexity of the models.

##### Peripersonal space

Similarly, to the analysis used for the BLT, for the baseline-corrected *audio-tactile RTs* (see above), we first checked if an effect of lesion lateralization (RBD/LBD) in patients exists: ‘lesion lateralization’ (RBD, LBD), ‘limb’ (affected, unaffected) and ‘distance’ (near, medium, far) were used as fixed factors. If a main effect or interaction of brain lesion emerged, RBD and LBD patients were considered separately for further comparisons with the control group, otherwise all patients were considered together. Then, to compare data obtained in patients and control participants, we used the factor ‘limb’ which levels were coded as nested (4 levels: affected/unaffected, belonging to the patients and left/right belonging to the controls) and ‘distance’ as the fixed factor.

#### Prevalence and multiple regressions

When a significant difference between patients and controls emerged at BLT and PPS tasks from LMM (i.e. the outcomes of interest, see Supplementary material), the following subsequent analyses were performed.

First, to evaluate the *prevalence of deficits in body and space representations*, scores from the two tasks were transformed into *z*-scores based on the corresponding values (mean, standard deviation) obtained in controls.^28^ Patients with *z*-scores ≥1.5 in one of the behavioural outcomes were classified as ‘potentially impaired’ in BLT or PPS.

Second, the outcomes of interest at the BLT and PPS task, as well as the sum of the affirmative answers at the ALEFq, were considered as the dependent variable in *multiple linear regressions* which was run to examine the relationship between *BR distortions* and patients’ clinical characteristics (e.g. level of motor, sensory or cognitive impairment, used as predictors). See Supplementary material for further details. The same approach has been used to explore possible links among the outcomes of interest at the BLT and PPS tasks and at the ALEFq, by running multiple regression analyses on the outcomes of one specific task (e.g. BLT) with the outcomes of the other tasks set as predictors (e.g. PPS, ALEFq).

#### Lesion analyses

BLT and PPS scores were used for lesions analyses to identify brain areas or connections associated with mBR and PPS deficits. Patients’ lesions were delineated using a semi-automated method,^58^ and used to compute subtraction maps (binary variable, impaired versus unimpaired patients), disconnection maps^59,60^ (binary variables) and voxel-based lesion-symptom mapping (VLSM,^58^ both continuous and binary variables). In addition, scores at ALEFq were used as the continuous variable for the VLSM (no comparisons with the control group could be performed on this questionnaire). Possible imbalance among the impaired and not impaired patients for the lesion extension, age and the severity of motor, sensory and cognitive impairment were checked through direct comparisons between the two groups (Mann–Whitney test) for lesion subtraction. Lesion volume was added as a regressor in the VLSM. Demographic (age), clinical (lesion size, sensory and motor deficits) and neuropsychological (neglect) data were considered in the models as control variables for the disconnection maps. Further details related to lesion analyses are reported in Supplementary material.

### Data availability

The data that support the findings of this study are available from the corresponding author, upon reasonable request.

## Results

### Implicit representation of the affected arm: BLT

Fifty-eight out of 60 patients were considered for the BLT, while two patients were excluded because of difficulty in maintaining the limb posture during the task. The 58 patients included for the BLT (mean: 56.4 ± 14, range: 23–81) were compared with 45 age-matched healthy controls (mean: 55.9 ± 22.4, range: 23–86, Mann–Whitney test: *P* = 0.28).

#### Estimated dimensions

We considered the *estimated dimension* of upper limbs, i.e. the ratio between the perceived and the real dimensions. No significant effects of lesion lateralization emerged (*R*^2^ = 0.80, *P*-values always higher than 0.18), thus all patients were compared with controls in the following analyses on the estimated dimensions (‘upper limb dimensions’: arm length, arm width, hand length, hand width and ‘limb’: affected/unaffected for patients, left/right for controls). The model (*R*^2^ = 0.60) comparing patients and controls on the estimated dimension reveals a significant interaction between ‘upper limb dimensions’ and ‘limb’ [*F*(9,721) = 9.58, *P* < 0.001]. To explore this interaction, we ran separate analyses for each ‘upper limb dimension’ with ‘limb’ as a factor.

The model on the *arm length* (*R*^2^ = 0.85) reveals a significant effect of ‘limb’ [*F*(3,118) = 3.58, *P* = 0.016; age: *P* = 0.005]. Patients perceived their affected arm as shorter than their unaffected one (*P* = 0.009), while no difference between the two arms was present in controls (*P* = 0.27).

**Figure 1:**
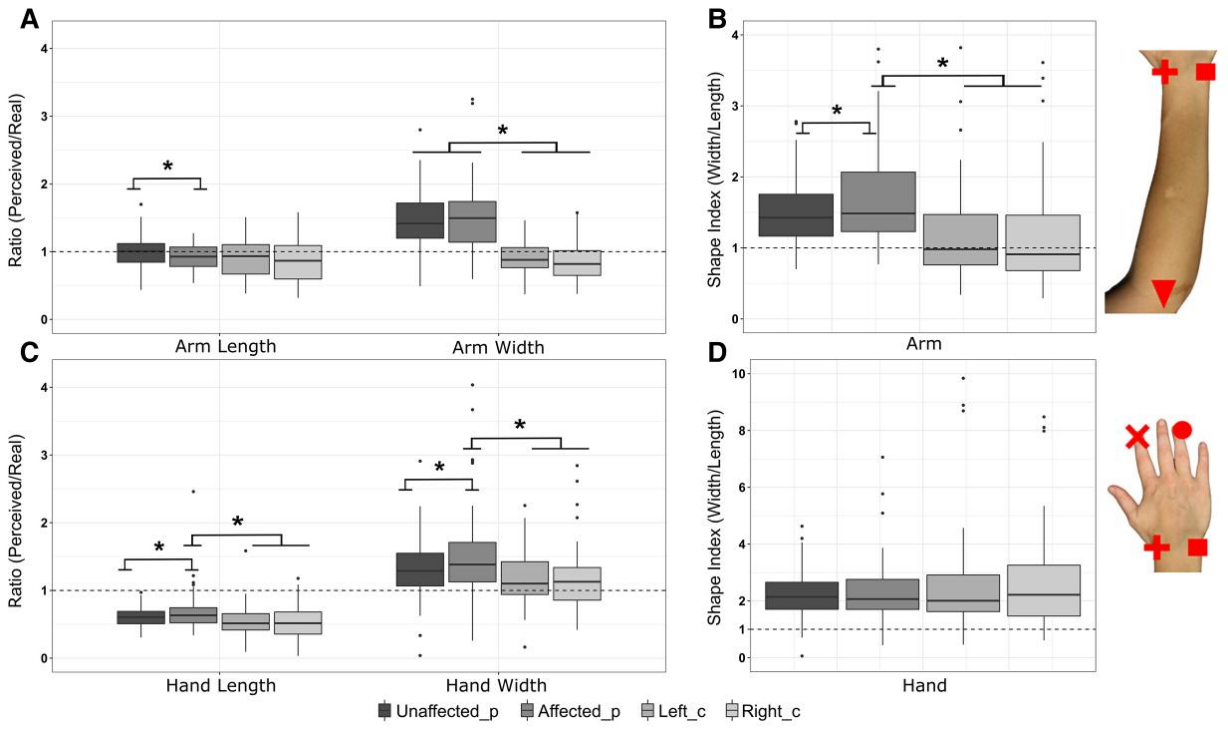
**Distortions in the perceived dimensions of the affected limb.** The boxplots [internal line for median, whiskers for the largest (upper) and the smallest (lower) value ≥ 1.5 ×  IQR, black dots for outliers] show the results at the BLT for the estimated dimension of the arm (**A**) and hand (**C**) in patients (*N* = 58, 21 females, Unaffected/Affected_p) and controls (*N* = 45, 34 females, Left/Right_c) [LMM: estimated dimensions: *F*(9,721) = 9.58, *P* < 0.001]. The dashed line set at 1 indicates the correspondence between perceived and real body part dimensions, with values <1 indicating an underestimation. The normalized shape index of the arm (**B**) and the hand (**D**), with values >1 indicating a higher estimated dimension on the width with respect to the length (distortions in global shape) [LMM: shape index: *F*(1,143) = 58.08, *P* < 0.0001]. Asterisks indicate significant differences in *post hoc* comparisons (all *P*-values <0.021) within and between-groups.

*Arm width* [*R*^2^ = 0.85, *F*(3,124) = 24.06, *P* < 0.00] was perceived larger in patients than controls (all *P*-values < 0.001), without significant difference between the two limbs (*P* = 0.61) (Fig. 1).

The models on the *hand length* [*R*^2^ = 0.76, *F*(3,130) = 4.38, *P* = 0.006; age: *P* = 0.32] and on the *hand width* [*R*^2^ = 0.72, *F*(3,136) = 4.02, *P* = 0.009] revealed that, in patients, the affected side was perceived as significantly longer (*P* = 0.009) and larger (*P* = 0.02) with respect to the unaffected one. The hand length and width asymmetries observed in patients were not present in controls, where the perceived dimensions of the left and right hand were comparable (all *P*-values > 0.71) (see Fig. 1).

Distortions in arm and hand width and length emerged between patients and controls. To integrate them in a unique model of upper limb shape perception in chronic stroke, we computed a normalized shape index, which is the ratio between the estimated width and the estimated length.^32^

#### Normalized shape index

We tested if an effect of patients’ brain lesion emerged on the *normalized shape index* (only data in patients, fixed factors: ‘lesion lateralization’, ‘body parts’, hand and arm). No significant difference was found between LBD and RBD patients (*R*^2^ = 0.75, all *P*-values >0.72), thus all patients were compared with controls in the following analyses on the normalized shape index. We excluded data from one control subject that turned out to be an outlier (+ 2 SD from the mean). Separate analyses for the hand and arm were performed, as the model (*R*^2^ = 0.73) showed an effect of ‘body parts’ [*F*(1,143) = 58.08, *P* < 0.0001; age: *P* = 0.006].

For the *arm*, the model [*R*^2^ = 0.85, *F*(3,119) = 5.53, *P* = 0.002; age: *P* = 0.03] revealed that the bias in the *normalized shape index* of the affected arm in patients was greater than the bias in the unaffected limb (*P* = 0.021) and the left (*P* = 0.017) or right (*P* = 0.019) limbs in controls (Fig. 1).

For the *hand*, the model revealed no significant difference between patients and controls [*R*^2^ = 0.75, *F*(3,130) = 0.67, *P* = 0.57], suggesting a similar global perceived shape in the two groups for both limb sides (see Fig. 1). Altogether, these results suggest a distortion in the global shape of the affected arm in chronic stroke patients, reflected by a reduced perceived length of the affected arm when compared with the unaffected one and an overestimation of the arm width.

Similar alterations were also found in an explicit task assessing metric representations of the upper limb, the AAT (see Supplementary results and Supplementary Fig. 3).

#### PPS: audio-tactile interaction task

Forty-three patients were able to perceive the tactile stimulus necessary for the PPS task, but only 39 were finally included in the analysis because of accuracy (see Supplementary material). The 39 stroke patients included for the PPS (mean: 56.7 ± 12.95, range: 23–80) were compared with 36 age-matched healthy controls (mean: 57.1 ± 23, range: 23–91, Mann–Whitney test: *P* = 0.25).

##### Multisensory PPS processing

Patients were slower than controls in responding to tactile stimuli, especially for the affected limb (see Supplementary results and Supplementary Fig. 1). To gather a measure of multisensory PPS, beyond the deficit in unisensory tactile processing, RTs to audio-tactile stimuli were corrected by subtracting the fastest mean RT among the unimodal tactile conditions from audio-tactile RTs (see above). The model on corrected audio-tactile RTs run in patients to test an effect of ‘lesion lateralization’ (fixed effects: ‘lesion lateralization’, ‘limb’: affected, unaffected, and ‘distance’: near, medium, far) did not reveal any significant difference [R^2^ = 0.91, *F*(1,40) = 2.14, *P* = 0.15; age: *P* = 0.54]. Thus, we considered the data of all patients together in the following comparisons with controls.

**Figure 2:**
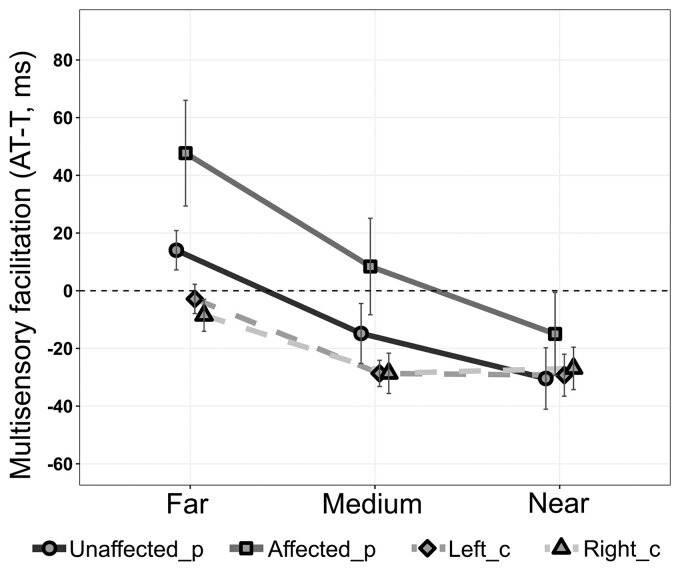
**Distortions in PPS representation**. The figure shows the results at the PPS task for all patients (*N* = 39, 13 females, Unaffected/Affected_p) and controls (*N* = 36, 25 females, Left/Right_C). Audio-tactile (AT) RTs (mean ± standard errors, ms) corrected for the unimodal tactile baseline (T, the fastest RTs to unimodal tactile stimulation, dashed line, 0, the baseline) (AT-T) are shown as a function of sound distance (far, medium and near). Negative values indicate a multisensory facilitation effect in responding to audio-tactile stimuli with respect to the unimodal tactile baseline. Corrected AT RTs for the affected side were significantly slower than those for the unaffected limb (*P* = 0.002) in patients and those for both limbs in controls (all *P*-values < 0.02) [LMM: *F*(3,192) = 6.061, *P* = 0.001].

The model run on corrected RTs comparing all patients and controls (factors: ‘limb’ and ‘distance’) (*R*^2^ = 0.58) shows a main effect of ‘distance’ [*F*(2,120) = 13.89, *P* < 0.001], indicating faster RTs when tactile stimuli were presented with near or medium sounds, rather than with far sounds (near versus far: *P* < 0.0001; medium versus far: *P* = 0.002; medium versus near: *P* = 0.26). Importantly, a significant effect of ‘limb’ also emerged [*F*(3,192) = 6.061, *P* = 0.001]. RTs for the affected side were significantly slower than those for the unaffected limb (*P* = 0.002) in patients and for both limbs in controls (all *P*-values < 0.02). RTs for the unaffected side were comparable to those of controls (all *P*-values < 0.74) (Fig. 2).

A further explorative analysis on the LBD (n = 17) and RBD (n = 22) patients considered separately is reported in Supplementary material (Supplementary Fig. 2), although not supported by any significant interaction with the factor ‘lesion lateralization’.

### Prevalence of body and space distortions

Considering the outcome of interests at the BLT (i.e. normalized shape index of the affected arm) and PPS task (i.e. audio-tactile RTs around the affected limb averaged between the three sounds distances), it emerges that:

- 39.5% of patients presented distortions (with respect to controls, see Supplementary material) in at least one task (15 out of 38 patients, considering the total of patients having available data at both tasks; 20 out of 60 patients (33.3%), considering all patients included in the study);
- 19% of patients were impaired at the BLT (11/58 patients) and 28.2% (11/39 patients) at the PPS task; among those patients showing distortions, two patients were impaired at both tasks, while the other patients were selectively impaired in one task, and not in the other (7/11 impaired only at the BLT and not at the PPS; 7/11 impaired only at the PPS task and not at the BLT; then two patients were impaired at the BLT and AAT and two at the PPS and the AAT, see Supplementary Table 1).

### Affected limb explicit feelings questionnaire

**Figure 3:**
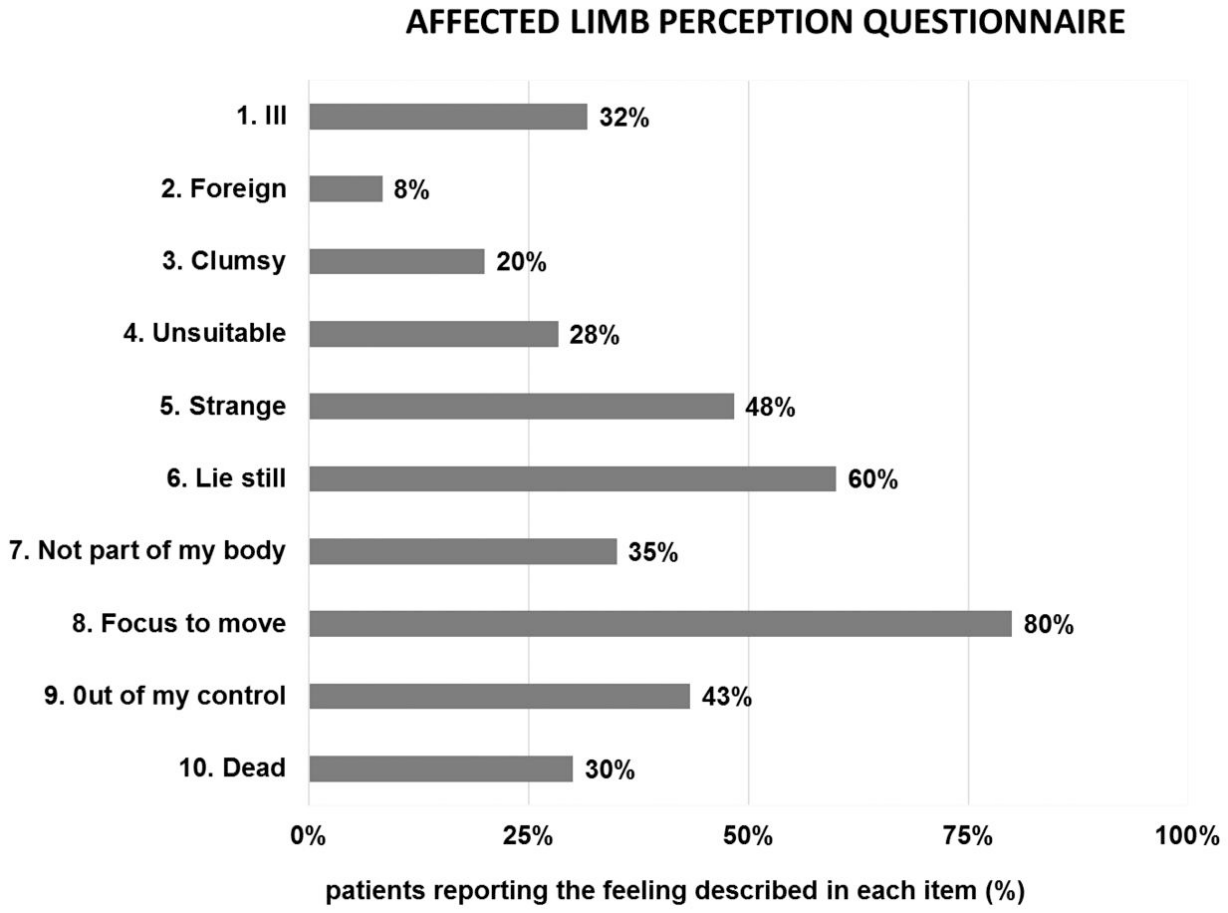
**Altered feelings towards the affected limb.** Percentage of patients (*N* = 60 in total, 21 females) reporting explicit positive answers at each item of the ALEFq.

All patients answered the ALEFq. They reported at least 2 (out of 10) negative feelings about their affected limb. The highest number of affirmative answers was 8, reported by two patients, while on average patients reported almost four feelings (mean = 3.85; standard deviation = 1.41). Thirty-five per cent of patients reported disownership and 43% loss of agency for their affected limb (see Fig. 3).

### Multiple regression analyses

Multiple linear regressions were run to examine the relationship between BR distortions and patients’ clinical characteristics, i.e. their level of sensory, motor and cognitive impairment. Hierarchical cluster analysis, applied to reduce the dimensionality of clinical data, highlighted (i) a cluster of neuropsychological scores reflecting a general measure of cognitive functioning; (ii) a cluster capturing the global level of motor impairment and (iii) two clusters of sensory deficits concerning, respectively, tactile acuity and proprioception for the first, and tactile sensibility for the second (see Supplementary material).

These clusters together with other patients clinical characteristics (age, time since stroke, the affected hemisphere, self-reported pain during motion obtained at the corresponding Fugl–Meyer subscale) were inserted as predictors in multiple regressions, while patient’s scores at BLT, PPS task and at the ALEFq represented the outcomes of interests.

**Figure 4:**
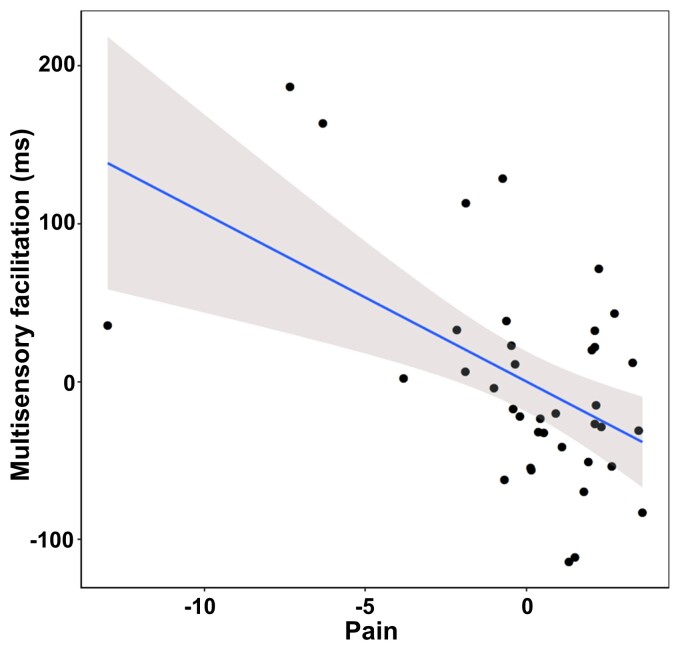
**Relation between results at the PPS task and clinical scores**. The partial regression plot (multiple regression analysis, see Supplementary material) on multisensory facilitation around the affected limb (PPS outcome of interest, higher RTs indicate less facilitation) and pain (self-reported pain scores at the Fugl–Meyer subscale, z-scores, lower values indicate greater reported pain).

The model for the BLT outcome (i.e. normalized arm shape index of the affected arm) was not significant (*P* = 0.55). Instead, the model on the PPS outcome (i.e. audio-tactile RTs around the affected limb averaged between the three sounds distances) [*R*^2^ = 0.41, *F*(8,29) = 2.57, *P* = 0.03] revealed a significant effect of pain during motion (*β* = −0.60, SE = 0.185, *P* = 0.003), indicating that patients who reported greater pain showed less multisensory facilitation in the audio-tactile task on the affected side (Fig. 4).

**Figure 5:**
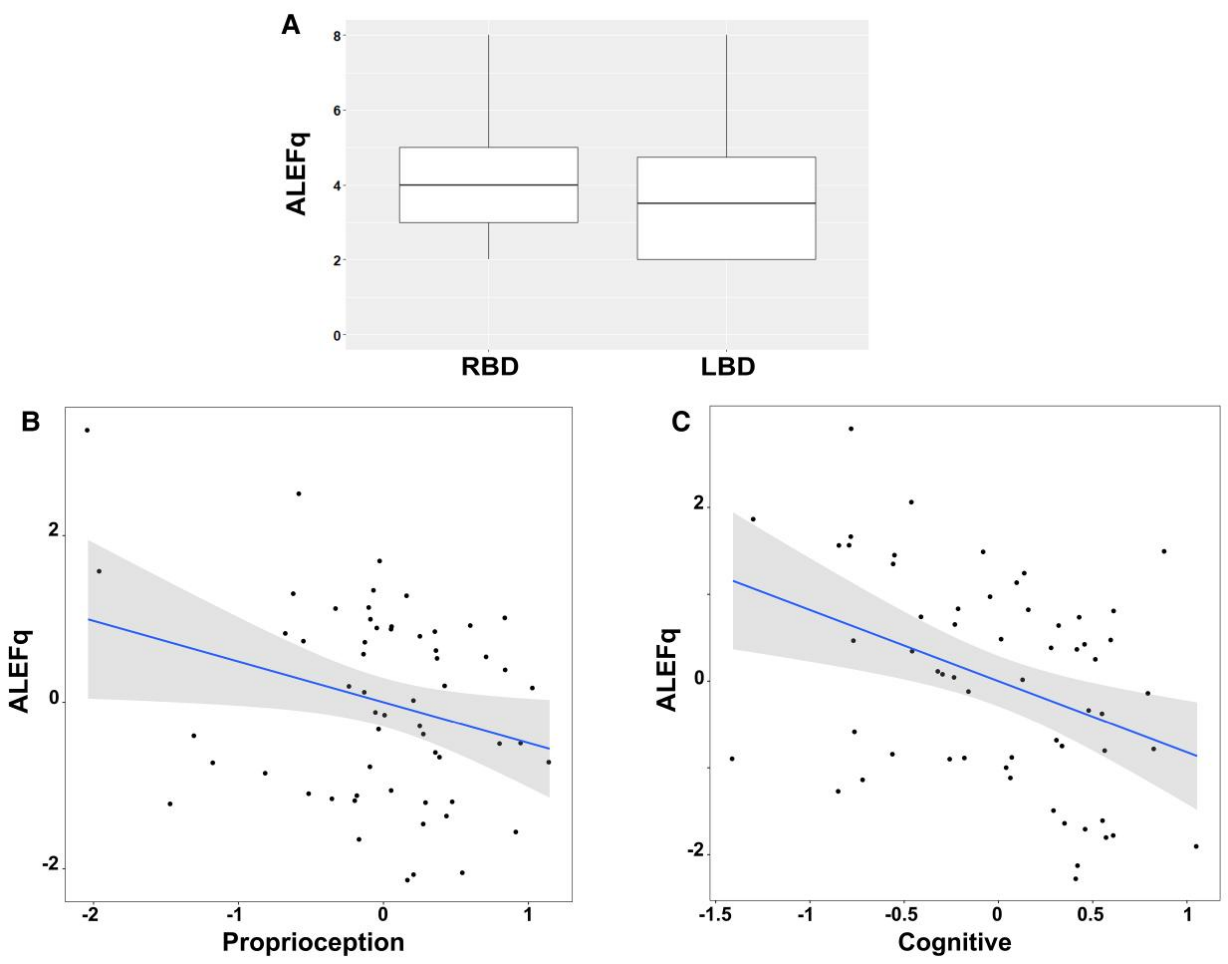
**Relation between results at the ALEFq and clinical scores**. The multiple regression analysis (see Supplementary material) on the ALEFq scores indicates that a higher number of altered feelings reported at the questionnaire correlate with RBDs (**A**), low proprioception (**B**) and cognitive disabilities (**C**). B and C are partial regression plots (z-scores, on the y-axis higher values indicate higher distortions, on the x-axis higher values indicate higher performances).

Moreover, the model was significant for the ALEFq (sum of the affirmative answers) [*R*^2^ = 0.291, *F*(8,50) = 2.57, *P* = 0.02] and indicates correlations with the sensory cluster related to proprioception (*β* = −0.32, SE = 0.16, *P* = 0.044), neuropsychological impairment (*β* = −0.40, SE = 0.14, *P* = 0.005) and the lesion lateralization (*β* = −0.66, SE = 0.27, *P* = 0.016), with more frequently reported altered feelings in RBD patients (Fig. 5).

Finally, no significant results emerged (all *P*-values >0.25) from multiple regression analyses run to explore the possible relationship between the outcomes of interest at the BLT, PPS and ALEFq.

### Lesion analyses

**Figure 6:**
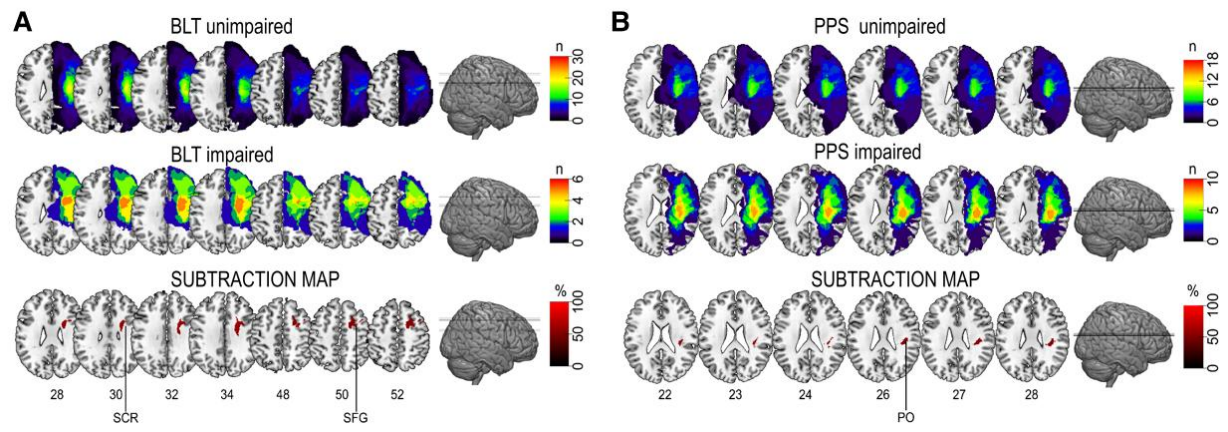
**Subtraction lesion analysis.** Images were superimposed on the ch256 template, the anatomical localization was assessed by using the Harvard–Oxford atlas for the cortical regions and the JHU atlas for the white matter. Lesion overlap among patients impaired (*n* = 6) and unimpaired (*n* = 30) at the BLT in SCR and the SFG (**A**) and among patients unimpaired (*n* = 18) and impaired (*n* = 10) at the PPS task, in the parietal operculum (PO) (**B**). The colour bars in first two lines indicate the number of overlapping lesions. The colour bars in the third line indicate the percentage of overlapping: a threshold of 50% is used for illustration purposes.

We first qualitatively compared brain lesions in patients with impaired versus non-impaired BR and PPS by using *subtraction analysis*. For the BLT, impaired patients showed more common lesions (50–60%) in the superior corona radiata (SCR) and the superior frontal gyrus (SFG) than patients with unimpaired BR (Fig. 6). The subtraction analysis for the PPS outcome indicated that the parietal operculum was more often affected in patients with impaired performance with a prevalence of around 50–60% (Fig. 6).

Two separate control analyses were run to ensure that the results in the lesion subtraction were not due to any imbalance in the lesion extension (number of the affected voxel), age or in the severity of motor, sensory and cognitive deficits between patients impaired and unimpaired respectively at BLT and at PPS. For these analyses, the same neuropsychological (1 cluster), motor (1 cluster) and sensory (2 clusters) clusters used for multiple regression analyses were considered. No significant differences in lesion extension, age, neuropsychological, motor and sensory deficits (Mann–Whitney test, a significant threshold set at 0.05/6 comparisons) were found between patients impaired and unimpaired at the BLT (all *P*-values >0.108) or the PPS (all *P*-values >0.113).

**Figure 7:**
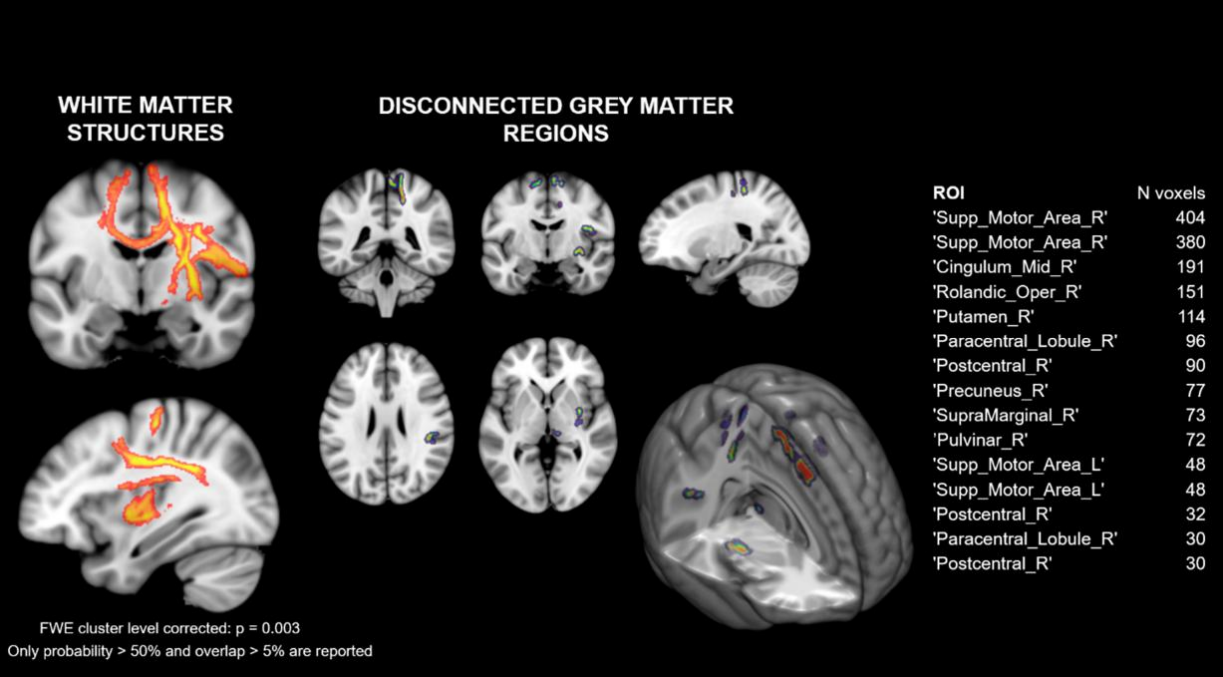
**Disconnection maps.** A set of white matter structures (left panel) and disconnected grey matter regions (right panel) were found to be associated with poorer performance at the PPS task in RBD patients. White matter structures (left) include the superior longitudinal fasciculus (I–II), the hand superior U tract, the corticospinal tract, the longitudinal segment of the arcuate fasciculus, the frontal commissural tract, the frontoinsular tract (IV–V) and the anterior thalamic projections in the right hemisphere; the superior longitudinal fasciculus I, the corticospinal tract, the cingulum and the anterior thalamic projections in the left hemisphere. Grey matter regions are listed in the table on the right.

We then applied *disconnection analyses* to search for neural correlates of mBR and PPS deficits at the network level. Disconnection analysis indicated a set of white matter structures and disconnected grey matter areas, mainly encompassing parieto-frontal regions, associated with poor multisensory facilitation assessed with the PPS task in RBD patients (see Fig. 7).

At a more liberal threshold (non-parametric *P* < 0.01 uncorrected), impairment on the BLT task in RBD patients was associated with lesion of the corticospinal tract and of transcallosal fibres connecting the right supplementary motor area and the left anterior cingulate cortex (Supplementary Fig. 4).

Further, *VLSM* did not reveal any significantly statistical results, likely because of the limited number and heterogeneity of the available data (see Supplementary results).

## Discussion

This study represents the first systematic, quantitative analysis of distortions in body and space representations in chronic stroke patients with motor deficits. The results reveal deficits involving the mBR, PPS and subjective feelings concerning the affected limb.

### Distortion in the perceived dimensions of the affected limb

Findings at the BLT task revealed differences in the length and width of the affected hand in patients, but not a significant modification in the global shape of the hand. This could lead to interpret the differences observed on the hand length and width as a shift in both dimensions, to maintain a global shape similar to the unaffected side. Indeed, similarly distorted hands’ dimension appeared in patients and controls, with the hands perceived as shorter and wider, in line with previous evidence obtained in healthy subjects by using different versions of the BLT.^33,35,54^

Interestingly, we found significant distortions in the implicit perceived dimensions of the affected arm in patients. This is characterized by an underestimation of the arm length, associated with an overestimation of the arm width. Arm length underestimation has been previously reported in healthy older adults^19,32^ possibly linked to a reduced use of the upper limb. Also, arm length underestimation has been reported in different patients’ populations, following either significant alterations of the physical body structure, as in amputees^51,52^ or in achondroplastic dwarfs,^61,62^ or brain damage (through a forearm bisection task).^29^

Our results are novel in showing a global distortion of the perceived arm shape in the two dimensions (width/length), with overestimation of the arm width, which is reminiscent of the increased perceived size of body parts in healthy participants after anaesthesia,^63^ after transcranial magnetic stimulation over the somatosensory cortex^64^ or in patients with complex regional pain syndrome.^65^ Several, non-mutually exclusive, factors might explain the present finding.

First, the observed distortions in mBR might be caused by alterations in the afferent signals from the body, due to the persistent motor and somatosensory deficits. Current views state that mBR presents stereotyped distortions also in healthy participants, due to the combination of peripheral (e.g. anisotropy of tactile receptive fields) and central (cortical magnification of body parts in the somatosensory homunculus) biases.^36,66^ However, these distortions are probably constantly corrected by online multimodal bodily inputs.^36,54,67^ Thus, contralesional motor deficits present in all patients, inducing a lack or dysfunctional usage (e.g. learned non-use)^68,69^ of the affected limb, may, in turn, compromise the necessary update of stored mBR. Similarly, the important deficits of proprioception and tactile sensibility present in our sample, i.e. 32% and 23% of patients, respectively (see Supplementary Table 2), might impact those mechanisms by limiting the role of sensory signals in updating mBR. Nevertheless, our multiple regression models are not conclusive in revealing a specific predictor. Note, however, that the lack of significant correlations between the observed distortions in BR and sensorimotor clinical scores could be due to the chronic phase of our assessment, likely masking possible earlier deficits and different recovery patterns.

Then, alterations in the perception of the affected arm could be linked to lesions in the putative areas underlying BR. A qualitative analysis based on lesions subtraction highlighted two areas more frequently affected in patients with versus patients without deficits in mBR: the SCR, crucial for arm representation,^70^ embodiment^20^ and for the brain–body bidirectional flow of information and the SFG, important in motor recovery of hand function after stroke.^71^ Together, these observations would support the hypothesis that distortions in mBR could arise from reduced sensorimotor information due to a possible role of limited functionality and use of the affected limb.

This interpretation seems also in line with the results observed at the disconnection analysis, indicating a possible association with lesions of the corticospinal tract and transcallosal fibres connecting the right supplementary motor area and the left anterior cingulate cortex (Supplementary Fig. S6). However, this conclusion should be taken with caution as results from the subtraction analysis were not confirmed by statistical comparisons of brain lesions through VLSM and disconnection analysis emerged only when a more liberal threshold was considered. This is likely due to the limited numbers of available data and the high variability in available brain scans, combining data from patients examined in the acute or chronic phase (see Supplementary material).^58,72^

More data on sensorimotor abilities and brain imaging from stroke patients longitudinally along the recovery phases are necessary to fully understand the origin of distortions in the perception of the affected limb.

### Alteration in PPS representation

A new finding from the present data is the distortion in PPS representation around the affected limb. Here, we conceptualized PPS as a multisensory–motor interface between one’s own body and the environment,^38–40^ characterized by facilitation of tactile processing (i.e. faster RT to a tactile stimulus on the body) by external stimuli (e.g. a sound) presented close rather than far from the stimulated body part. Thus, we used an audio-tactile interaction task^32,41,42,50,55^ to test at which distance from the body a sound can induce a significant facilitation of tactile processing. Patients presented an overall reduced multisensory facilitation in tactile processing when sounds were presented on the contralesional side with respect to their unaffected side or with respect to healthy controls. Specifically, while baseline-corrected RTs were significantly speeded up at the near and medium distance in controls and only at the near distance in patients for stimuli on the ipsilesional side (see Supplementary analysis on bimodal RTs against the baseline), this effect was absent for the contralesional side. On the contralesional side, tactile processing was never facilitated by concurrent auditory stimuli, despite a distance-dependent speeding up of RTs, which could reflect a general expectancy effect,^73^ already visible in unimodal RTs (see Supplementary Fig. S1). This is in contrast with previous studies showing preserved audio-tactile multisensory facilitation in stroke patients^74^ with tactile extinction^75^ or pathological embodiment.^76^ However, in those studies, patients were selected for a specific characteristic of multisensory processing or altered bodily experience, while in the present study, patients were included because of a motor deficit, and their alterations in processing multisensory stimuli in space or body perception were investigated.

The explanation of this PPS alteration might lie in the link between PPS representation and the potential to act in space. A previous study reported difficulties of stroke patients (specifically RBD when compared with LBD) in performing a reachability judgement for stimuli in the reachable space.^77,^^78^ Although the notion of PPS and reachable space do not coincide,^79^ the presence of deficits in these spatial tasks after stroke clearly indicates a problem in processing multisensory cues for actions. Motor impairments due to stroke could reduce the possibility to act in space with the affected limb, thus altering the representation of PPS around that body part, resonating with previous results in healthy participants after 10 h of immobilization.^50^ This is in line with the notion of a link between PPS representation and the motor system.^38,80–84^ Finally, the impairment of PPS representation could be even more evident in RBD patients, where the occurrence of hemispatial neglect is more common.^15^ This latter hypothesis is supported by an additional analysis on RBD patients only (see Supplementary material) revealing significant multisensory facilitation effects due to near sound on the affected limb only in RBD patients without neglect. However, this result has to be considered with caution, given the limited number of patients with clear signs of neglect (*n* = 6) at the time of testing (i.e. chronic phase, potentially underestimating neglect symptoms in the current sample).

Multiple regression analyses did not show a direct link between PPS distortions in our sample and the level of motor impairment, probably because of the presence of significant motor impairments in all patients. However, a relationship between PPS alterations and pain during motion (Fugl–Meyer subscale) was found, suggesting that motion-related pain could be an additional detrimental factor, further limiting the use of the affected arm.

Thus, we propose that the limited use of the affected limb, possibly exacerbated by the presence of pain during movements, contributed to alterations in PPS representation. Qualitative analysis (subtraction) suggests that this deficit was more frequently associated with the lesion of the parietal operculum, which has been related with the processing of multiple bodily signals, including pain,^85^ and is considered a key region for PPS representation.^86^ Moreover, the explorative analysis run in RBD patients to identify a probabilistic map of disconnections between brain regions associated with the lack of multisensory facilitation on the affected arm^59,60^ indicated disconnections between brain regions considered critical for PPS representation in humans and monkeys, such as the parietal operculum,^87^ the cingulate cortex and the putamen.^86^ These areas are connected by well-defined parieto-frontal white matter tracts within the longitudinal fasciculus,^88^ which are the tracts more likely associated with PPS distortions from our disconnection analyses. The lack of statistical results at the VLSM and the limited numerosity and heterogeneity of the available lesions indicate that further studies are necessary to ultimately demonstrate that the alteration in PPS processing is linked to specific lesions in the parieto-frontal network involved in processing multisensory cues in space for action.

### Subjective perception of the affected limb

Our findings also revealed a high prevalence of persistent alterations in the subjective feeling of the contralesional limb as emerging from the ALEFq, not only in items assessing awareness of motor deficits (e.g. ‘I feel my limb is ill’), but also in those expressing more complex experiences of disownership (‘I feel my limb as foreign’) or lack of agency (‘My limb sometimes moves involuntarily, without my control’). The questionnaire has been partially adapted from studies in patients with complex regional pain syndrome^89,90^ where authors reported a high frequency of negative feelings in long-lasting disease, if explicitly assessed. These considerations seem particularly valuable in the clinical management of stroke patients, where feelings related to the affected limb may affect the therapeutic benefits of neurorehabilitation^15,27,91^, but are not assessed systematically. Interestingly, the present multiple regression analyses reveal that the ratings at the ALEFq were associated with a prevalence of RBD, proprioceptive deficits and generally lower cognitive functions. In line with this, other alterations of BR after stroke such as delusional disownership of the affected limb, such as in somatoparaphrenia^6,8,13^ and pathological embodiment,^19,20^ are typically associated with RBD and severe proprioceptive deficits. Our data support the link between altered BR, RBD and proprioceptive deficits along the different phases of the disease.

### Study limitations

The present work has potential limitations. First, the available clinical data refer only to the chronic phase, when the experimental tasks were administered, preventing the assessment of potential relationships between the observed bodily and space distortions and acute deficits or recovery dynamics. Second, we did not include patients without motor deficits, because the participants were recruited in the context of a study focused on motor recovery.^53^ Thus, the dissociation between motor deficits and metric BR/PPS deficits cannot be fully assessed. Finally, the limited number of available lesions, and their heterogeneity in terms of both method (CT, MRI) and time of the acquisition (i.e. different phases of the disease) may limit the interpretability of the anatomical results and may contribute to the absence of significant effects from VLSM.

### General conclusion and potential clinical implications

We report a pattern of body and space distortions of the affected upper limb in chronic stroke patients characterized by: (i) an alteration of the arm perceived dimension, (ii) reduced multisensory facilitation for stimuli presented in the PPS around the affected limb and (iii) altered feelings towards the affected limb. More than one-third (39.5%) of patients were impaired in at least one task. The present data reveal that, if systematically assessed, distortions in body and space representations are also common in chronic stroke patients. This has potential clinical implications in motivating quantitative assessments of body and space representations after stroke in earlier and later phases of the disease. Those distortions could have an impact on patient’s everyday life by influencing the spontaneous use of the affected limb and the effect of treatments.^53^ In this vein, monitoring distortions in body and space representations longitudinally could also clarify which clinical deficits and patients’ characteristics are associated with those distortions. This in turn would contribute to design tailored rehabilitative interventions, targeting sensorimotor functions or potentially, body and space representations, with the final aim of boosting functional recovery.

## Supplementary Material

fcac179_Supplementary_Data

## Abbreviations

AAT =: avatar adjustment task
AIC =: Akaike’s information criterion
ALEFq =: affected limb explicit feelings questionnaire
BIC =: Bayesian information criterion
BLT =: body-landmarks localization tasks
BR =: body representations
LBD =: left brain damage
LMM =: linear mixed model
mBR =: metric body representation
PPS =: peripersonal space
RBD =: right brain damage
RT =: reaction time
SCR =: superior corona radiata
SFG =: superior frontal gyrus
VLSM =: voxel-based lesion-symptom mapping

## References

[1] Ramsey LE , Siegel JS, Lang CE, Strube M, Shulman GL, Corbetta M. Behavioural clusters and predictors of performance during recovery from stroke. Nat Hum Behav. 2017;1(3):0038. 28713861 10.1038/s41562-016-0038 PMC5508212

[2] Go AS , Mozaffarian D, Roger VL, et al Heart disease and stroke statistics—2013 update. Circulation. 2013;127(1):e6–e245. 23239837 10.1161/CIR.0b013e31828124ad PMC5408511

[3] Langhorne P , Coupar F, Pollock A. Motor recovery after stroke: A systematic review. Lancet Neurol. 2009;8(8):741–754. 19608100 10.1016/S1474-4422(09)70150-4

[4] Mullick AA , Subramanian SK, Levin MF. Emerging evidence of the association between cognitive deficits and arm motor recovery after stroke: A meta-analysis. Restor Neurol Neurosci. 2015;33(3):389–403. 26410581 10.3233/RNN-150510 PMC4923759

[5] Carlsson H , Rosén B, Pessah-Rasmussen H, Björkman A, Brogårdh C. SENSory re-learning of the UPPer limb after stroke (SENSUPP): Study protocol for a pilot randomized controlled trial. Trials. 2018;19(1):229. 29665842 10.1186/s13063-018-2628-1 PMC5904984

[6] Romano D , Maravita A. The dynamic nature of the sense of ownership after brain injury. Clues from asomatognosia and somatoparaphrenia. Neuropsychologia. 2019;132(June):107119. 31194981 10.1016/j.neuropsychologia.2019.107119

[7] Jenkinson PM , Moro V, Fotopoulou A. Definition: Asomatognosia. Cortex. 2018;101:300–301. 29510834 10.1016/j.cortex.2018.02.001

[8] Ronchi R , Bassolino M, Viceic D, et al Disownership of body parts as revealed by a visual scale evaluation. An observational study. Neuropsychologia. 2020;138:107337. 31923525 10.1016/j.neuropsychologia.2020.107337

[9] Vallar G , Ronchi R. Somatoparaphrenia: A body delusion. A review of the neuropsychological literature. Exp Brain Res. 2009;192:533–551. 18813916 10.1007/s00221-008-1562-y

[10] Gerstmann J . Problem of imperception of disease and of impaired body territories with organic lesions: Relation to body scheme and its disorders. Arch Neurol Psychiatry. 1942;48(6):890–913.

[11] Feinberg TE , Venneri A. Somatoparaphrenia: Evolving theories and concepts. Cortex. 2014;61(1953):74–80. 25481466 10.1016/j.cortex.2014.07.004

[12] Pacella V , Foulon C, Jenkinson PM, et al Anosognosia for hemiplegia as a tripartite disconnection syndrome. Elife. 2019;8:e46075. 31383259 10.7554/eLife.46075 PMC6684265

[13] Moro V , Besharati S, Scandola M, et al The Motor Unawareness Assessment (MUNA): A new tool for the assessment of Anosognosia for hemiplegia. J Clin Exp Neuropsychol. 2021;43:91–104. 33588707 10.1080/13803395.2021.1876842

[14] Bisiach E , Vallar G, Perani D, Papagno C, Berti A. Unawareness of disease following lesions of the right hemisphere: Anosognosia for hemiplegia and anosognosia for hemianopia. Neuropsychologia. 1986;24(4):471–482. 3774133 10.1016/0028-3932(86)90092-8

[15] Caggiano P , Jehkonen M. The ‘Neglected’ personal neglect. Neuropsychol Rev. 2018;28(4):417–435. 30547412 10.1007/s11065-018-9394-4 PMC6327000

[16] Schwoebel J , Coslett HB. Evidence for multiple, distinct representations of the human body. J Cogn Neurosci. 2005;17(4):543–553. 15829076 10.1162/0898929053467587

[17] Razmus M . Body representation in patients after vascular brain injuries. Cogn Process. 2017;18(4):359–373. 28852890 10.1007/s10339-017-0831-8 PMC5688204

[18] Di Vita A , Palermo L, Boccia M, Guariglia C. Topological map of the body in post-stroke patients: Lesional and hodological aspects. Neuropsychology. 2019;33(4):499–507. 30730163 10.1037/neu0000536

[19] Garbarini F , Fossataro C, Berti A, et al When your arm becomes mine: Pathological embodiment of alien limbs using tools modulates own body representation. Neuropsychologia. 2015;70:402–413. 25448852 10.1016/j.neuropsychologia.2014.11.008

[20] Pia L , Fossataro C, Burin D, et al The anatomo-clinical picture of the pathological embodiment over someone else’s body part after stroke. Cortex. 2020;130:203–219. 32679408 10.1016/j.cortex.2020.05.002

[21] Garbarini F , Fossataro C, Pia L, Berti A. What pathological embodiment/disembodiment tell us about body representations. Neuropsychologia. 2020;149:107666. 33130159 10.1016/j.neuropsychologia.2020.107666

[22] Errante A , Rossi Sebastiano A, Ziccarelli S, et al Structural connectivity associated with the sense of body ownership: a diffusion tensor imaging and disconnection study in patients with bodily awareness disorder. Brain Commun. 2022;4(1):fcac032. 35233523 10.1093/braincomms/fcac032 PMC8882004

[23] Bassolino M , Bouzerda-Wahlen A, Moix V, et al You or me? Disentangling perspectival, perceptual, and integrative mechanisms in heterotopagnosia. Cortex. 2019;120:212–222. 31330470 10.1016/j.cortex.2019.05.017

[24] de Langavant L C , Trinkler I, Cesaro P, Bachoud-Lévi AC. Heterotopagnosia: When I point at parts of your body. Neuropsychologia. 2009;47(7):1745–1755. 19397870 10.1016/j.neuropsychologia.2009.02.016

[25] Hammerbeck U , Gittins M, Vail A, Paley L, Tyson SF, Bowen A. Spatial neglect in stroke: Identification, disease process and association with outcome during inpatient rehabilitation. Brain Sci. 2019;9(12):374. 10.3390/brainsci9120374 PMC6956021 31847166

[26] Farne A , Buxbaum L, Ferraro M, et al Patterns of spontaneous recovery of neglect and associated disorders in acute right brain-damaged patients. J Neurol Neurosurg Psychiatry. 2004;75(10):1401. 15377685 10.1136/jnnp.2002.003095 PMC1738754

[27] Gialanella B , Monguzzi V, Santoro R, Rocchi S. Functional recovery after hemiplegia in patients with neglect: The rehabilitative role of anosognosia. Stroke. 2005;36(12):2687–2690. 16269649 10.1161/01.STR.0000189627.27562.c0

[28] van Stralen HE , Dijkerman HC, Biesbroek JM, et al Body representation disorders predict left right orientation impairments after stroke: A voxel-based lesion symptom mapping study. Cortex. 2018;104:140–153. 28732749 10.1016/j.cortex.2017.05.025

[29] Tosi G , Romano D, Maravita A. Mirror box training in hemiplegic stroke patients affects body representation. Front Hum Neurosci. 2018;11:617. 29354040 10.3389/fnhum.2017.00617 PMC5758498

[30] Longo MR . Distortion of mental body representations. Trends Cogn Sci. 2022;26:241–254. 34952785 10.1016/j.tics.2021.11.005

[31] Bassolino M , Serino A. Representation and perception of the body in space. In: Encyclopedia of Behavioral Neuroscience, Vol. 2-3, 2nd edn. Academic Press; 2021:640–656.

[32] Sorrentino G , Franza M, Zuber C, Blanke O, Serino A, Bassolino M. How ageing shapes body and space representations: A comparison study between healthy young and older adults. Cortex. 2021;136:56–76. 33460913 10.1016/j.cortex.2020.11.021

[33] Longo MR . The effects of instrumental action on perceptual hand maps. Exp Brain Res. 2018;236(11):3113–3119. 30132042 10.1007/s00221-018-5360-x

[34] de Vignemont F . Body schema and body image—Pros and cons. Neuropsychologia. 2010;48(3):669–680. 19786038 10.1016/j.neuropsychologia.2009.09.022

[35] Peviani V , Bottini G. The distorted hand metric representation serves both perception and action. J Cogn Psychol. 2018;30(8):880–893.

[36] Longo MR , Haggard P. An implicit body representation underlying human position sense. Proc Natl Acad Sci USA. 2010;107(26):11727–11732. 20547858 10.1073/pnas.1003483107 PMC2900654

[37] Tamè L , Longo MR. Probing the neural representations of body-related stimuli: Comment on “Revealing the body in the brain: An ERP method to examine sensorimotor activity during visual perception of the body-related information” by Alejandro Galvez-Pol, Beatriz Calvo-Merino. Cortex. 2021;134:358–361. 33012526 10.1016/j.cortex.2020.08.019

[38] Serino A . Peripersonal space (PPS) as a multisensory interface between the individual and the environment, defining the space of the self. Vol. 99. Elsevier; 2019:138–159. 10.1016/j.neubiorev.2019.01.016 30685486

[39] Cléry J , Ben Hamed S. Frontier of self and impact prediction. Front Psychol. 2018;9:1073. 29997556 10.3389/fpsyg.2018.01073 PMC6030567

[40] Bufacchi RJ , Iannetti GD. An action field theory of peripersonal space. Trends Cogn Sci. 2018;22:1076–1090. 30337061 10.1016/j.tics.2018.09.004 PMC6237614

[41] Serino A , Noel J-P, Galli G, et al Body part-centered and full body-centered peripersonal space representations. Sci Rep. 2015;5(1):1–11. 10.1038/srep18603 PMC4686995 26690698

[42] Canzoneri E , Magosso E, Serino A. Dynamic sounds capture the boundaries of peripersonal space representation in humans. PLoS One. 2012;7(9):3–10. 10.1371/journal.pone.0044306 PMC3460958 23028516

[43] De Vignemont F , Serino A, Wong HY, Farnè A. The world at our fingertips: A multidisciplinary exploration of peripersonal space. Oxford University Press; 2021.

[44] Martel M , Cardinali L, Roy AC, Farnè A. Tool-use: An open window into body representation and its plasticity. Cogn Neuropsychol. 2016;33(1-2):82–101. 27315277 10.1080/02643294.2016.1167678 PMC4975077

[45] Bassolino M , Serino A, Ubaldi S, Làdavas E. Everyday use of the computer mouse extends peripersonal space representation. Neuropsychologia. 2010;48(3):803–811. 19931547 10.1016/j.neuropsychologia.2009.11.009

[46] Serino A , Bassolino M, Farnè A, Làdavas E. Extended multisensory space in blind cane users. Psychol Sci. 2007;18(7):642–648. 17614874 10.1111/j.1467-9280.2007.01952.x

[47] Miller LE , Montroni L, Koun E, Salemme R, Hayward V, Farnè A. Sensing with tools extends somatosensory processing beyond the body. Nature. 2018;561(7722):239–242. 30209365 10.1038/s41586-018-0460-0

[48] Maravita A , Iriki A. Tools for the body (schema). Trends Cogn Sci. 2004;8(2):79–86. 15588812 10.1016/j.tics.2003.12.008

[49] Galigani M , Castellani N, Donno B, et al Effect of tool-use observation on metric body representation and peripersonal space. Neuropsychologia. 2020;148:107622. 32905815 10.1016/j.neuropsychologia.2020.107622

[50] Bassolino M , Finisguerra A, Canzoneri E, Serino A, Pozzo T. Dissociating effect of upper limb non-use and overuse on space and body representations. Neuropsychologia. 2014;70:385–392. 25462198 10.1016/j.neuropsychologia.2014.11.028

[51] Canzoneri E , Marzolla M, Amoresano A, Verni G, Serino A. Amputation and prosthesis implantation shape body and peripersonal space representations. Sci Rep. 2013;3(1):2844. 24088746 10.1038/srep02844 PMC3789144

[52] Rognini G , Petrini FM, Raspopovic S, et al Multisensory bionic limb to achieve prosthesis embodiment and reduce distorted phantom limb perceptions. J Neurol Neurosurg Psychiatry. 2019;90:833–836. 30100550 10.1136/jnnp-2018-318570 PMC6791810

[53] Crema A , Bassolino M, Guanziroli E, et al Neuromuscular electrical stimulation restores upper limb sensory-motor functions and body representations in chronic stroke survivors. Med. 2022;3(1):58–74.e10. 35590144 10.1016/j.medj.2021.12.001

[54] Longo MR , Haggard P. Implicit body representations and the conscious body image. Acta Psychol (Amst). 2012;141:164–168. 22964057 10.1016/j.actpsy.2012.07.015

[55] Canzoneri E , Ubaldi S, Rastelli V, Finisguerra A, Bassolino M, Serino A. Tool-use reshapes the boundaries of body and peripersonal space representations. Exp Brain Res. 2013;228(1):25–42. 23640106 10.1007/s00221-013-3532-2

[56] Fuentes CT , Longo MR, Haggard P. Body image distortions in healthy adults. Acta Psychol (Amst). 2013;144(2):344–351. 23933684 10.1016/j.actpsy.2013.06.012

[57] Noel J-PP , Pfeiffer C, Blanke O, Serino A. Peripersonal space as the space of the bodily self. Cognition. 2015;144:49–57. 26231086 10.1016/j.cognition.2015.07.012 PMC4837893

[58] de Haan B , Karnath H-O. A hitchhiker’s guide to lesion-behaviour mapping. Neuropsychologia. 2018;115:5–16. 29066325 10.1016/j.neuropsychologia.2017.10.021

[59] Thiebaut De Schotten M , Tomaiuolo F, Aiello M, et al Damage to white matter pathways in subacute and chronic spatial neglect: A group study and 2 single-case studies with complete virtual “in vivo” tractography dissection. Cereb Cortex. 2014;24(3):691–706. 23162045 10.1093/cercor/bhs351

[60] Foulon C , Cerliani L, Kinkingnéhun S, et al Advanced lesion symptom mapping analyses and implementation as BCBtoolkit. Gigascience. 2018;7:1–17. 10.1093/gigascience/giy004 PMC5863218 29432527

[61] Cimmino RL , Spitoni G, Serino A, et al Plasticity of body representations after surgical arm elongation in an achondroplasic patient. Restor Neurol Neurosci. 2013;31(3):287–298. 23396370 10.3233/RNN-120286

[62] Di Russo F , Committeri G, Pitzalis S, et al Cortical plasticity following surgical extension of lower limbs. Neuroimage. 2006;30(1):172–183. 16288893 10.1016/j.neuroimage.2005.09.051

[63] Gandevia SC , Phegan CMLL. Perceptual distortions of the human body image produced by local anaesthesia, pain and cutaneous stimulation. J Physiol. 1999;514(2):609–616. 9852339 10.1111/j.1469-7793.1999.609ae.x PMC2269086

[64] Giurgola S , Pisoni A, Maravita A, Vallar G, Bolognini N. Somatosensory cortical representation of the body size. Hum Brain Mapp. 2019;40(12):3534–3547. 31056809 10.1002/hbm.24614 PMC6865590

[65] Moseley GL . Distorted body image in complex regional pain syndrome. Neurology. 2005;65(5):773. 16157921 10.1212/01.wnl.0000174515.07205.11

[66] Tamè L , Azañón E, Longo MR. A conceptual model of tactile processing across body features of size, shape, side, and spatial location. Front Psychol. 2019;10(February):291. 30863333 10.3389/fpsyg.2019.00291 PMC6399380

[67] Taylor-Clarke M , Jacobsen P, Haggard P. Keeping the world a constant size: Object constancy in human touch. Nat Neurosci. 2004;7(3):219–220. 14966526 10.1038/nn1199

[68] Taub E , Uswatte G, Mark VW, Morris DM. The learned nonuse phenomenon: Implications for rehabilitation. Eura Medicophys. 2006;42(3):241–255. 17039223

[69] Carey LM , Matyas TA, Baum C. Effects of somatosensory impairment on participation after stroke. Am J Occup Ther. 2018;72(3):7203205100p1-7203205100p10. 10.5014/ajot.2018.025114 PMC5915232 29689179

[70] Morecraft RJ , Herrick JL, Stilwell-Morecraft KS, et al Localization of arm representation in the corona radiata and internal capsule in the non-human primate. Vol. 125. Oxford University Press; 2002. 10.1093/brain/awf011 11834603

[71] Rehme AK , Fink GR, Von Cramon DY, Grefkes C. The role of the contralesional motor cortex for motor recovery in the early days after stroke assessed with longitudinal fMRI. Cereb Cortex. 2011;21(4):756–768. 20801897 10.1093/cercor/bhq140

[72] Karnath HO , Rennig J. Investigating structure and function in the healthy human brain: Validity of acute versus chronic lesion-symptom mapping. Brain Struct Funct. 2017;222(5):2059–2070. 27807627 10.1007/s00429-016-1325-7

[73] Kandula M , Van der Stoep N, Hofman D, Dijkerman HC. On the contribution of overt tactile expectations to visuo-tactile interactions within the peripersonal space. Exp Brain Res. 2017;235(8):2511–2522. 28528459 10.1007/s00221-017-4965-9 PMC5502056

[74] Bolognini N , Russo C, Vallar G. Crossmodal illusions in neurorehabilitation. Front Behav Neurosci. 2015;9(August):212. 26321933 10.3389/fnbeh.2015.00212 PMC4530305

[75] Làdavas E , di Pellegrino G, Farnè A, Zeloni G. Neuropsychological evidence of an integrated visuotactile representation of peripersonal space in humans. J Cogn Neurosci. 1998;10(5):581–589. 9802991 10.1162/089892998562988

[76] Fossataro C , Bruno V, Bosso E, et al The sense of body-ownership gates cross-modal improvement of tactile extinction in brain-damaged patients. Cortex. 2020;127:94–107. 32171114 10.1016/j.cortex.2020.02.004

[77] Bartolo A , Carlier M, Hassaini S, Martin Y, Coello Y. The perception of peripersonal space in right and left brain damage hemiplegic patients. Front Hum Neurosci. 2014;8:3. 24478670 10.3389/fnhum.2014.00003 PMC3902828

[78] Shahvaroughi-Farahani A , Linkenauger SA, Mohler BJ, Behrens SC, Giel KE, Karnath H-O. Body size perception in stroke patients with paresis. PLOS ONE. 2021;16:e0252596. 34086777 10.1371/journal.pone.0252596 PMC8177542

[79] Zanini A , Patané I, Blini E, et al Patterns of multisensory facilitation distinguish peripersonal from reaching space. bioRxiv. 2020:2020.06.01.127282.

[80] Cooke DF , Taylor CSR, Moore T, Graziano MSA. Complex movements evoked by microstimulation of the ventral intraparietal area. Proc Natl Acad Sci USA. 2003;100(10):6163–6168. 12719522 10.1073/pnas.1031751100 PMC156343

[81] Finisguerra A , Canzoneri E, Serino A, Pozzo T, Bassolino M. Moving sounds within the peripersonal space modulate the motor system. Neuropsychologia. 2014;70:421–428. 25281311 10.1016/j.neuropsychologia.2014.09.043

[82] Graziano MSA , Cooke DF. Parieto-frontal interactions, personal space, and defensive behavior. Neuropsychologia. 2006;44(13):2621–2635. 17128446 10.1016/j.neuropsychologia.2005.09.011

[83] Makin TR , Holmes NP, Brozzoli C, Farnè A. Keeping the world at hand: Rapid visuomotor processing for hand-object interactions. Exp Brain Res. 2012;219(4):421–428. 22526949 10.1007/s00221-012-3089-5

[84] Serino A , Annella L, Avenanti A. Motor properties of peripersonal space in humans. PLoS One. 2009;4(8):e6582. 19668366 10.1371/journal.pone.0006582 PMC2719059

[85] Eickhoff SB , Schleicher A, Zilles K, Amunts K. The human parietal operculum. I. Cytoarchitectonic mapping of subdivisions. Cereb Cortex. 2006;16(2):254–267. 15888607 10.1093/cercor/bhi105

[86] Grivaz P , Blanke O, Serino A. Common and distinct brain regions processing multisensory bodily signals for peripersonal space and body ownership. Neuroimage. 2017;147(iii):602–618. 28017920 10.1016/j.neuroimage.2016.12.052

[87] Bernasconi F , Noel J-PP, Park HD, et al Audio-tactile and peripersonal space processing around the trunk in human parietal and temporal cortex: An intracranial EEG study. Cereb Cortex. 2018;28(9):3385–3397. 30010843 10.1093/cercor/bhy156 PMC6095214

[88] Matelli M , Luppino G. Parietofrontal circuits for action and space perception in the macaque monkey. NeuroImage. 2001;14:S27–S32. 11373129 10.1006/nimg.2001.0835

[89] Förderreuther S , Sailer U, Straube A. Impaired self-perception of the hand in complex regional pain syndrome (CRPS). Pain. 2004;110(3):756–761. 15288417 10.1016/j.pain.2004.05.019

[90] Galer BS , Jensen M. Neglect-like symptoms in complex regional pain syndrome: Results of a self-administered survey. J Pain Symptom Manage. 1999;18(3):213–217. 10517043 10.1016/s0885-3924(99)00076-7

[91] Spaccavento S , Cellamare F, Falcone R, Loverre A, Nardulli R. Effect of subtypes of neglect on functional outcome in stroke patients. Ann Phys Rehabil Med. 2017;60(6):376–381. 28958616 10.1016/j.rehab.2017.07.245

