# Supplementary material for: Body and peripersonal space representations in chronic stroke patients with upper limb motor deficits
Source: Brain Commun. 2022 Aug 5;4(4):fcac179. doi: 10.1093/braincomms/fcac179 (PMC9356734; doi:10.1093/braincomms/fcac179)
Supplement: fcac179_Supplementary_Data [file fcac179_supplementary_data.pdf]

SUPPLEMENTARY MATERIALS

Body and peripersonal space representations in chronic stroke patients with upper limb motor deficits

Bassolino M.[1,2,3], Franza M.[1], Guanziroli E.[4], Sorrentino G.[1], Canzoneri E.[1], Colombo M.[4], Crema A.[5, 6, 7], Bertoni T.[2], Mastria G.[2], Vissani M.[1], Sokolov A.A.[8, 9], Micera S.[5,10], Molteni F.[4], Blanke O.[1,6*], Serino A.[1,2*]

*These authors contributed equally to the work

[1]Laboratory of Cognitive Neuroscience, Center for Neuroprosthetics and Brain Mind Institute, School of Life Science, Swiss Federal Institute of Technology (EPFL), Geneva, Switzerland
[2]Department of Clinical Neuroscience, Centre Hospitalier Universitaire Vaudois (CHUV), MySpace Lab, Lausanne, Switzerland
[3]Institute of Health, School of Health Sciences, HES-SO Valais-Wallis, Sion, Switzerland
[4]Department of Rehabilitation "Villa Beretta", Valduce Hospital, Costa Masnaga, Como, Italy
[5]Bertarelli Foundation Chair in Translational Neuroengineering, Centre for Neuroprosthetics and Institute of Bioengineering, School of Engineering, École Polytechnique Fédérale de Lausanne (EPFL), Lausanne, Switzerland.
[6]AGO Neurotechnologies, sàrl, Geneva, Switzerland.
[7]Wellcome Centre for Human Neuroimaging, Institute of Neurology, University College London (UCL), London, WC1N 3BG, UK
[8]Service de Neurologie, Département des Neurosciences Cliniques, Centre Hospitalier Universitaire Vaudois (CHUV), 1011, Lausanne, Switzerland
[9]The Biorobotics Institute and Department of Excellence in Robotics and AI, Scuola Superiore Sant'Anna, Pisa, Italy
[10]Department of Clinical Neuroscience, University of Geneva Medical School, Geneva, Switzerland

CONTENTS

1. SUPPLEMENTARY METHODS 3

1.1. Supplementary tables 1 and 2 3

1.2. Implicit body representation task: the body-landmarks localization task (BLT) 6

1.3. Peripersonal space task (PPS). 7

1.4. Affected limb explicit feelings questionnaire (ALEFq). 7

1.5. Explicit body representation task: the avatar adjustment task (AAT). 8

1.6. Clinical scales 9

1.7. Data analyses 9

Main statistical analysis: linear mixed models (LMM): general considerations. 9

Incidence of the body and space distortions. 10

Multiple linear regressions and hierarchical clustering 10

Lesion delineation and analysis. 11

2. SUPPLEMENTARY RESULTS 12

2.1 Peripersonal Space: audito-tactile interaction task. 12

2.2 Explicit body representation task: the avatar adjustment task (AAT) 16

2.3 Prevalence, multiple regression and lesion analyses about the AAT. *19*

2.4 Hierarchical clustering 19

2.5 Supplementary figures for results reported in the main text 20

2.6 Supplementary results in lesion analyses 20

3. SUPPLEMENTARY DISCUSSION 21

Distortions in the perceived metric characteristics of the affected upper limb captured by the AAT 21

4. SUPPLEMENTARY REFERENCES 23

1. SUPPLEMENTARY METHODS

1.1. Supplementary tables 1 and 2

ID	Age	G	Education	Time since stroke	Affected side	Stroke type	MRI	Lesion analysis	MI tot	MMSE	BLT	PPS	AAT
**1**	59	M	11	7.4	R	H	✓	✓	45	26	-	-	-
**2**	73	F	5	6.1	R	I	✓	✓	1	18	-	-	-
**3**	45	M	18	~1 year	R	I	✓	✓	40	25	-	-	**+**
**4**	55	F	11	~1 year	R	H	✓	✓	1	18	-	**+**	-
**5**	64	M	13	3.2	L	H	✓	✗	1	24	**+**	-	-
**6**	67	M	5	1.5	R	I	✓	✓	77	27	-	-	-
**7**	62	F	18	7.7	L	I	✗	✗	29	24	-	//	-
**8**	49	M	8	9.3	L	I	✓	✓	34	26	-	**+**	-
**9**	73	M	5	2.5	L	I	✓	✓	24	18	-	//	-
**10**	61	M	13	3.7	L	I	✗	✗	24	27	-	-	-
**11**	61	M	8	7.4	L	I	✓	✓	73	28	-	-	-
**12**	53	M	8	5.0	R	H	✓	✓	24	//	-	**+**	**+**
**13**	68	M	11	6.5	L	I	✗	✗	29	25	-	//	-
**14**	43	F	13	1.8	L	I	✓	✓	45	24	-	-	**+**
**15**	81	M	5	2.5	L	I	✗	✗	40	26	-	//	-
**16**	60	F	13	6.1	R	H	✓	✓	73	21	-	-	**+**
**17**	74	M	13	1.3	R	I	✗	✗	51	27	-	-	-
**18**	66	F	5	3.1	L	H	✓	✗	77	23	-	-	-
**19**	57	M	13	4.6	L	I	✓	✓	51	27	**+**	-	**+**
**20**	76	F	5	2.5	L	I	✗	✗	73	24	-	-	**+**
**21**	64	M	8	>10 years	L	I	✓	✓	24	26	//	//	**+**
**22**	77	M	3	3.7	L	I	✓	✓	48	26	-	**+**	-
**23**	56	M	13	>10 years	R	H	✗	✗	73	//	**+**	-	-
**24**	47	M	18	5.2	R	I	✓	✓	77	27	//	**+**	-
**25**	55	M	18	~1 year	R	I	✓	✓	84	26	-	//	-
**26**	26	F	8	~1 year	L	H	✓	✓	10	29	**+**	-	-
**27**	57	F	13	~1 year	L	H	✓	✗	50	29	**+**	-	-
**28**	57	M	13	2.5	R	I	✓	✓	45	13	-	-	-
**29**	52	F	8	3.1	L	I	✗	✗	67	29	-	**+**	-
**30**	53	M	13	1.5	R	I	✓	✓	77	29	-	-	-
**31**	43	F	17	6.6	L	I	✓	✓	19	27	-	//	-
**32**	67	M	8	~1 year	L	I	✓	✓	73	24	-	-	-
**33**	61	M	11	1.4	R	I	✗	✗	77	28	-	//	**+**

**34**	65	F	13	>10 years	R	I	×	×	48	26	+	//	-
**35**	52	F	18	4.1	R	I	✓	✓	62	26	-	-	-
**36**	66	M	10	2.5	L	H	✓	✓	81	28	-	+	+
**37**	80	M	5	2.6	L	I	×	×	66	21	-	-	-
**38**	36	F	13	4.1	R	I	✓	✓	67	29	-	-	-
**39**	26	M	11	10.0	R	I	✓	×	65	25	+	//	+
**40**	71	F	13	4.9	L	I	✓	✓	77	27	+	//	-
**41**	61	M	5	1.1	L	I	×	×	64	28	-	//	-
**42**	53	F	8	5.4	R	I	×	×	71	14	-	-	-
**43**	59	M	12	6.6	L	H	✓	✓	39	27	-	+	-
**44**	65	M	8	9.6	L	H	✓	✓	81	22	+	+	+
**45**	41	M	13	1.1	R	H	✓	✓	40	//	-	+	-
**46**	56	F	18	8.5	L	I	×	×	50	25	-	-	-
**47**	31	F	13	7.7	L	H	✓	✓	45	27	-	//	-
**48**	67	M	17	~1 year	L	I	✓	✓	24	24	+	//	-
**49**	48	M	18	2.7	L	I	✓	✓	19	27	-	-	-
**50**	47	F	18	>10 years	R	I	✓	✓	77	28	-	//	-
**51**	23	M	10	6.7	R	H	✓	✓	67	26	+	+	+
**52**	41	M	11	7.8	L	I	✓	✓	77	27	-	-	-
**53**	62	M	13	>10 years	R	I	✓	✓	45	20	-	-	-
**54**	49	M	13	3.1	R	I	✓	✓	60	25	-	//	-
**55**	24	F	13	~1 year	R	I	✓	✓	56	//	-	//	-
**56**	39	M	8	1.2	L	I	×	×	67	27	-	//	-
**57**	59	M	13	~1 year	L	I	×	×	73	28	-	//	+
**58**	63	M	8	~1 year	L	I	×	×	58	27	-	//	+
**59**	74	F	11	~1 year	R	I	×	×	67	27	-	//	-
**60**	63	M	8	~1 year	L	I	✓	✓	71	24	-	-	+

Supplementary table 1. *Patients' demographic and clinical data. ID= patient ID, Age (years), G= gender, Education (years), Time since stroke (years), Affected side (R= right, for left brain damaged patients, L=left, for right brain damaged patients), Stroke type (H = haemorrhagic, I = ischemic), MRI (× indicates that a CT and not an MRI was available), Lesion analysis (× indicates patients not included in the lesion analysis), MI total (total score of the Motricity Index), MMSE (corrected scores of Mini-Mental State Examination). All patients where chronic, i.e. more than 6 months from stroke onset ("~1year" indicates from more 6 months till 1 year). The individual performance at the behavioural tasks (BLT = body-landmarks localization task, PPS = peripersonal-space, and the AAT = avatar adjustment task) has been classified as "pathological" (+) when it exceeded 1.5 sd of the control group performance. // indicates missing data.*

ID	FM UL	ARAT TOT	MRC	Bisiach sensibility	Tactile acuity upper limb	Proprioception forearm	RAVEN ES	FAB ES	GO/NOGO ES latency	GO/NOGO ES accuracy
**1**	9	0	15	3	10	1	3	3	0	4
**2**	4	0	0	1	//	2	2	0	0	4
**3**	9	0	9	3	7	2	2	2	2	2
**4**	4	0	1	1	18	2	3	0	1	1
**5**	4	0	0	1	14	1	0	1	//	//
**6**	29	55	35	3	6	1	4	4	3	0
**7**	8	0	11	0	6	2	1	0	0	0
**8**	17	0	15	0	10	0	4	4	4	0
**9**	9	0	6	1	12	1	0	0	0	3
**10**	9	0	7	1	//	1	0	2	1	0
**11**	21	33	32	3	16	1	2	0	0	4
**12**	13	3	8	2	12	2	4	//	0	1
**13**	15	4	23	3	13	2	2	0	1	0
**14**	24	6	21	3	6	2	2	3	2	3
**15**	17	1	16	0	//	2	0	0	1	0
**16**	27	29	36	0	//	1	1	3	2	4
**17**	13	18	25	3	6	2	4	2	2	4
**18**	25	41	34	1	11	0	2	0	0	1
**19**	30	18	21	3	12	2	4	3	0	4
**20**	20	17	31	2	21	2	1	0	0	0
**21**	15	0	12	3	12	2	4	3	0	0
**22**	18	11	14	1	19	0		2	0	0
**23**	20	26	33	3	9	2	1	//	0	0
**24**	28	51	33	3	8	2	4	3	3	2
**25**	34	56	36	3	10	2	1	0	1	2
**26**	4	0	2	3	14	2	2	4	0	0
**27**	7	0	16	3	14	2	4	3	3	3
**28**	11	5	9	3	18	0	0	//	//	//
**29**	29	53	31	2	16	2	4	4	0	3
**30**	34	54	34	3	12	2	1	0	0	0
**31**	8	4	4	2	16	1	1	0	0	0
**32**	35	40	41	3	10	2	1	0	0	0
**33**	29	48	33	3	7	1	4	4	4	2
**34**	28	31	27	3	20	2	2	1	1	3
**35**	27	10	31	3	7	2	4	0	4	3
**36**	36	57	37	3	21	2	3	4	0	3
**37**	27	23	25	3	7	2	3	4	4	3
**38**	34	40	32	3	9	2	4	3	1	3
**39**	23	56	24	3	13	2	2	1	2	2
**40**	36	57	33	3	//	2	2	2	3	2

**41**	11	6	26	3	12	2	4	3	1	2
**42**	18	13	21	3	9	2	3	0	0	0
**43**	15	8	16	3	14	0	2	1	1	2
**44**	32	48	32	3	11	2	2	4	1	2
**45**	20	2	28	0	//	0	//	//	//	//
**46**	19	4	14	2	18	2	2	3	1	0
**47**	22	6	22	1	16	1	4	4	0	3
**48**	10	0	6	2	17	1	3	3	1	3
**49**	4	0	6	3	10	2	3	4	1	3
**50**	36	57	36	3	6	2	2	4	4	3
**51**	29	35	22	3	6	2	2	0	0	3
**52**	34	46	30	3	12	2	2	3	1	2
**53**	23	12	17	3	9	2	4	0	4	0
**54**	16	20	25	3	4	2	3	//	3	2
**55**	22	4	16	0	21	1	4	//	//	//
**56**	34	46	28	3	11	2	4	1	4	3
**57**	36	45	32	3	14	2	3	4	1	3
**58**	23	5	25	3	8	2	2	4	4	3
**59**	26	8	28	3	16	2	0	0	0	3
**60**	25	51	30	3	18	1	2	3	2	3

Supplementary table 2. *Patients detailed characteristics. ID= patient ID, FM-UL (Fugl-Meyer upper limb subscale), ARAT tot (total score of Action Research Arm Test), MRC (total score of Medical Research Council)[1,2], tactile sensibility [3], tactile acuity (two-points discrimination threshold)[4] for the forearm, proprioception (Joint Position Sense)[5] for the forearm (also the arm and the hand were evaluated for both tactile acuity and proprioception, but they are not reported in the table for sake of brevity), RAVEN (Raven's progressive matrices)[6], FAB (Frontal assessment battery) [7], GO/NO GO accuracy and latency (Battery TEA, was considered but not reported in the table for sake of brevity)[8–10], ES = equivalent score.*

1.2 Implicit body representation task: the body-landmarks localization task (BLT)

Task description. This task is briefly described in the main text, while further details are provided here. During the task, participants were seated on a chair with the forearm (affected or unaffected, depending on the condition) resting palm down on a table, aligned with the shoulder and positioned at around 20 cm far from the body midline and with the elbow joint at around 8-10 cm far from the border of the table. To avoid movements and to standardize as much as possible the position, the participants' forearm was fixed to the table for the entire duration of the experiment while the hand was resting on a not working computer mouse. The actual position of the five anatomical landmarks was recorded before starting the experiment, while the participant was blindfolded. Afterward, a wooden table (80 cm x 80 cm, 20 cm of height) was positioned above the participant's arm and an additional cloth was used to occlude the view of the shoulders. Then, the blindfold was removed, and the experimenter manually moved a marker attached to a wooden stick over the surface of the table, along the longitudinal axis of the forearm. Participants were instructed to verbally indicate when the marker was perceived just above the felt position of the target anatomical landmark. This task can be

considered implicit because participants have to indicate only the location of some anatomical landmarks, while not providing explicit judgments about width or length of the body parts[11]. On each trial, the experimenters (EG/GS) showed on their body the target landmark to judge. At the patient's verbal signal, the experimenter stopped the movement, leaving the marker where indicated, while participants were allowed to further adjust the position of the marker by asking the experimenter to move it again (backward, forward or laterally). Following the final confirmation, the marker's location was recorded. The task comprised five blocks in which we recorded the five landmarks, randomized within each block, for a total of five repetitions for each landmark. Data were collected for both left and right or affected/unaffected upper limb in randomized order among participants. We used retro-reflective markers (1 cm of diameter) recorded using an optical motion capture system (OptiTrack V120: TRIO; Motive 1.7.5 Final 64-bit, 2015) and a custom-made script in Matlab (R2018a).

1.2. Peripersonal space task (PPS).

Task description. This task is briefly described in the main text, while further details are provided here. During the audio-tactile interaction task used to evaluate PPS representation around the hand (e.g. [12–18]) participants were blindfolded and seated in a comfortable position with the tested arm (left or right, affecting or unaffected, depending on the condition) resting on a table. For the tactile stimulation, we used a single vibrotactile device that was placed on the dorsum of participant's hand (Precision MicroDrives shaftless vibration motors, model 312– 101, 3 V, 60 mA, 9000 rpm, 150 Hz, 5 g). Tactile stimulation lasted 100 ms. The acoustic stimuli consisted of a dynamic broadband noise looming toward participants' hand. Two loudspeakers generated the sound: one was positioned near the participant's hand (0 cm), and the other one placed 100 cm distant from the near speaker (i.e., far from the participants' hand). To give the impression of approaching to the subject's body, the sound was manipulated in intensity[17]. Acoustic and tactile stimuli were delivered in a controlled manner using an in- house software (ExpyVR, https://c4science.ch/w/expyvr-wiki/installation/).

In total, the trials consisted of 20 bimodal trials for each temporal delays (20x3), in which participants heard a sound and at a given moment in time they received the tactile stimulation; 10 unimodal trials for each temporal delays (10x3), in which the tactile stimulation was delivered in the absence of auditory stimulation. In addition, 20 catch trials with only auditory stimuli were included to control for automatic motor response. During each trial, the sound lasted 3 s and participants had 2 s, starting from the moment in which the tactile stimulus was delivered, to answer to the stimulation. Inter-stimulus intervals were randomized between 0.5, 0.75 and 1 s. Both sides (left/right or affected/unaffected) were tested in patients and controls in randomized order.

1.3. Affected limb explicit feelings questionnaire (ALEFq).

Description. To assess the presence of explicit disturbances in upper limb perception, such as feelings of disembodiment, numbness or foreignness, we adapted items from two previously proposed questionnaires for patients with chronic pain, i.e. the Feeling of foreignness questionnaire[19] and the Neurobehavioral Questionnaire[20]. Five questions propose adjectives related to the limb (e.g. clumsy, unsuitable, from the Feeling of foreignness questionnaire[19]) and the other 5

items describe feelings such as dis-ownership or loss of agency (from the Neurobehavioral Questionnaire[20], see Supplementary table 3 for a detailed description). Patients had to positively answer to the items if they experienced the described sensations about their affected limb.

Parameters of interest. We calculate the percentage of patients reporting affirmative answers to each item. In addition, for each patient, we computed a total score by summing all the affirmative answers, so that a higher score indicates a higher number of reported altered feelings[21].

**Affected limb explicit feelings questionnaire (ALEFq)**
Items from Feeling of foreignness questionnaire (adapted from Forderreuther et al., 2004)<br><br>I feel as my affected arm is:<br>1. Ill<br>2. Foreign<br>3. Clumsy<br>4. Unsuitable<br>5. Strange
Items from Neurobehavioral Questionnaire (adapted from Galer & Jensen, 1999)<br><br>6. If I don't focus my attention on my limb it would lie still, like dead weight<br>7. My limb feels as though it is not part of the rest of my body<br>8. I need to focus all of my attention on my limb to make it move the way I want it to<br>9. My limb sometimes moves involuntarily, without my control<br>10. My limb feels dead to me

Supplementary table 3. *The ten items of the "Affected limb explicit feelings questionnaire" (ALEFq). Patients have to indicate if they experience the sensations described in each of the 10 items.*

1.4. Explicit body representation task: the avatar adjustment task (AAT).

Task description. This task aims to explicitly assess the perceived dimension of the participants' upper limbs with respect to a visual model[15]. During the AAT, participants were seated in front of a monitor (52X32cm) with a white cloth covering their shoulders, arms, and hands. A body model on an avatar was showed in diagonal view. The avatar respected anatomical characteristics of the general population on the basis of the age and gender[22] and it was set to match each participant's age and gender. In each trial, participants were instructed to modify the dimensions (i.e., the width or the length) of the observed avatar's hands or arms to resemble their occluded body parts. To do so, they had to verbally guide the experimenter who operated on a keyboard to adjust the avatar's body parts (i.e. arms or hands) dimensions, making them longer or shorter, fatter, or narrower. The starting dimension of the body model was extremely enlarged in one-half of the trials and extremely shrunk in the other half. Divided into two blocks, we recorded four trials for each dimension (width and length, two trials with each starting position) and each body parts (arm and hand), for both left and right side, for a total of 32 trials (16 for each block). As for the BLT, data were collected for both left and right or affected/unaffected upper limb in randomized order among participants.

The avatars were designed by using an open-source tool capable of producing realistic virtual humans (Make Human,

http://www.makehumancommunity.org/frontpage/makehuman_110_has_been_released.html/).

Parameters of interest. We considered parameters similar to the BLT: the *estimated dimension*, that is the estimated length and width of the arm and the hand, expressed as a percentage of overestimation or underestimation with respect to the average (i.e., unbiased) size of the body model (i.e., the size corresponding to the reference population of equal age and gender provided by the software, see[22,23]). Similarly, to the BLT, values above 1 indicated an overestimation and values below 1 indicated an underestimation with respect to the average size of the reference population (=1). We calculated the mean of the data collected in all trials for each dimension and body parts (arm length, arm width, hand length, hand width). Finally, as in the BLT, we calculated the *Normalized Shape Index,* a general index of the perceived shape of the arm and the hand, as the ratio between the estimated dimension on the width and the length. As defined above, a value higher than 1 indicates that participants showed a higher estimated dimension for the width than for the length. Prevalence, multiple regression and lesion analyses about the AAT were also run with the same methodology applied for BLT and PPS and are reported below.

1.5. Clinical scales

Patients' motor abilities were evaluated through the following clinical scales (see Supplementary table 2): the Motricity Index and the Medical Research Council (MRC), assessing individual muscles strengths; the Action Research Arm Test (ARAT) and the Blocks and Box task assessing functional recovery; the Motor Activity Log (MAL), assessing usage of the upper limb in everyday life and the Fugl-Meyer, assessingmotor and sensory functioning, balance, range of motion and pain. In addition, the Modified Ashworth Scale (MAS) has been also used to control for patients' spasticity(e.g.[1,2]).

Moreover, sensory deficits were evaluated in terms of tactile acuity (two-points discrimination thresholds[4]), tactile sensitivity (standard procedure to evaluate somato-sensory impairment[3]) and proprioception (joint position test[5]).

Finally, a neuropsychological assessment including tests evaluating executive functions (Frontal assessment battery, FAB[7]), inhibitory control (go-no go test of the Battery TEA, latency and accuracy[9]), general cognitive impairment (Mini mental state examination, MMSE[24]) and reasoning (Raven's progressive matrices[6]) was considered. In addition, hemispatial neglect was assessed in RBD patients by using the Bell cancellation test (asymmetry score) and the visual field/neglect subtest of the Test of Everyday Attention (TEA, latency and accuracy scores, asymmetry score[8–10]). Equivalent scores (from 0 to 4) were computed when possible (Raven, FAB, go-no latency and accuracy), while in the other cases, we considered scores corrected for age and education (MMSE).

1.6. Data analyses

Main statistical analysis: linear mixed models (LMM): general considerations.

We test whether in patients body representations of the affected and unaffected limb were different and if these diverge from those of a group of age-matched healthy controls. The main statistical procedures run on the scores captured at the BLT and the PPS task have been described in the main text. For the AAT, the same analyses described for the BLT were

used. Briefly, first, we test in patients a main effect or interaction of patients' brain lesion by using brain "lesion lateralization" (RBD, LBD), "limb" (affected, unaffected) and "upper limbs dimensions" (arm length, arm width, hand length, hand width) as factors. If a main effect or interaction of brain lesion emerged, RBD and LBD patients were considered separately for further comparisons with the control group, otherwise all patients were considered together. Then, data obtained in patients and control participants were compared, by using upper limb dimensions (arm length, arm width, hand length, hand width) and "limb" (4 levels: affected, unaffected, left, right), as factors. Participants were considered as random effect, together with additional random effects inserted on the basis of a model selection with AIC and BIC criteria, while the age of participants was inserted as covariate in all models, in line with previously showed age-related effects in body representations (and in particular at the AAT[23]). For fixed effects, p-values were obtained by likelihood ratio tests, and degree of freedom were approximated using Satterthwaite method. To explore significant interactions, follow up analyses or Tukey post hoc test were used. The details of each analysis are reported in the main text.

Incidence of the body and space distortions.

We compared individual patients' data from the distribution of healthy controls (see Methods) to gather a measure of prevalence of a deficit in each of the function considered. To do that, for each behavioral task, we selected those indexes where a significant comparison with the age-matched control group emerged in the main analysis (linear mixed model). To understand the incidence of possible distortions at the BLT, and the audio- tactile (PPS) tasks, as well as for the AAT (results for the AAT are reported in Supplementary results), the outcome measures capturing the performance of patients in each behavioral task were transformed into z-scores based on the corresponding values (mean, standard deviation) obtained in the age-matched control group in the same task (e.g.[9]). Patients with z-scores≥1.5 in one of each behavioral outcome will be labelled as "impaired" in that specific task, because higher values indicate a higher magnitude of the distortion. All the calculations were performed with the software R (R Core Team, 2017, http://www.R-project.org/).

Multiple linear regressions and hierarchical clustering

We then investigated whether deficits in BR were linked to patients' motor, sensory and cognitive functions. To this aim, multiple linear regressions were performed to examine the relationship between the outcomes of interest, i.e. patients scores at BLT, audio-tactile (PPS) tasks and at the ALEFq, and some clinical and autobiographical predictors. As for the analysis on the incidence of the distortions, we used as a dependent variable for each behavioral task (BLT, PPS task, as well as AAT), those indexes where a significant comparison with the age-matched control group emerged in the main analysis (linear mixed model). In addition, the total score (sum of the affirmative answers) at the ALEFq was considered. All the outcomes were transformed to z-scores and used as continuous variables. Higher scores indicate a higher distortion. As predictors, we included patients' characteristics, i.e. the age (in years), the time since stroke (in log-transformed days) and the affected hemisphere (binary variable), self-reported pain scores obtained at the corresponding subscale of Fugl-Meyer assessment scale, as well as scores acquired with clinical scales on motor, sensory and cognitive functions (see above). First, we transformed all numerical scales to z-scores and imputed missing values using the averages of the 10 nearest neighbors within the same category of predictors: motor scales (no missing values),

sensory scales (5.5% missing values), neuropsychological scales (6.6% missing values) ("knnImputation" function in R). If needed, values were changed in sign so that for all variables higher values indicate higher performances. Considering that for the behavioral outcomes higher scores indicate higher distortions, we expect negative correlation with predictors. Then, to reduce data dimensionality, we applied hierarchical clustering ("hclust" package in R) on each category of predictors (see below, hierarchical cluster analysis). The results were analyzed case by case to obtain meaningful indexes from the predictors (see results). The obtained indexes were thus used as predictors in separate multiple linear regressions ("lm" function from base package in R) on each outcome of interests (see above). Please note that we report a conservative result after removing one high leverage subject from the regression on the ALEFq (as observed in the diagnostic plot). The results ofthe regression without the exclusion of this participant are in the same direction and even more significant.

Lesion delineation and analysis.

Lesion delineation. The patients' lesions were delineated by using a semi-automated method based on the Clusterize SPM toolbox (SPM 12). The Clusterize algorithm automatically clustered the radiological image (CT or MRI), and then the experimenter manually selects those clusters corresponding to the lesions (e.g.[25]). Each neurological image was normalized (or segmented and normalized, when high resolution radiological images were available) using the Clinical Toolbox implemented in the software SPM 12[26]. The normalization allowed the translation of each lesion map to a standard MRI template for subsequent analyses.

We used the radiological images performed for clinical purposes in acute or chronic phase. The semi-automated method was performed by some authors (MF, MB, GS) and then the final selected lesions were reviewed by an additional author, expert neurologist (AAS).

Subtraction maps. Subtraction maps between the group of patients with impaired and unimpaired performance at one of the behavioural outcomes of interests (BLT, PPS and as well as AAT) were computed in MRIcron (Chris Rorden, University of South Carolina, Columbia, SC, USA). For this purpose, we considered as behavioural outcomes one index per each behavioural task, BLT, PPS and as well as AAT. We selected those indexes were a significant comparison with the age-matched control group emerged in the main analysis (linear mixed model). To maximize the number of patients in each group, subtraction maps was obtained by considering only flipped (on the right) images.

Voxel-based lesion- symptom mapping (VLSM). Then, to link the behavioural outcomes of interest to specific damaged voxels, separate *voxel-based lesion- symptom mapping (VLSM)* analyses for each behavioural parameter was performed using the software NiiStat (https://www.nitrc.org/projects/niistat). The same behavioural outcomes considered for the subtraction maps were both used here as continuous or binary variables (impaired versus unimpaired patients). In addition, scores at ALEFq was used as continuous variable (no comparisons with the control group could be performed on this test). The analyses were performed with general linear model (least squares' linear regression) with FDR (false discovery rate) correction for multiple comparisons, with a statistical threshold of $p< 0.05$ and by taking into consideration only voxels damaged in at least the 10% of the sample. Lesion volume was used as regressor.

Disconnection maps. Finally, we applied an advanced lesion analysis method[27] based on probabilistic map of disconnections from each patient's brain lesion to identify the disconnections between brain areas that could be associated with body and space distortions at the group level (e.g. http://www.bcblab.com/BCB/Disconnectome.html). We computed the disconnection between brain areas using the BCBtoolkit[27]. This approach uses a dataset of 10 healthy controls[28] diffusion weighted imaging to track fibers passing through each lesion. First, patients' lesions were normalized to the MNI152 template. For each participant tractography was then estimated as indicated in[29]. Patients' lesions in the MNI152 space were registered to each control native space using affine and diffeomorphic deformations and subsequently used as seed for the tractography in Trackvis[30]. Tractographies from the lesions were transformed in visitation maps, binarized and brought to the MNI152 using the inverse of the precedent deformations. Then, we produced a percentage overlap map by summing at each point in MNI space the normalized visitation map of each healthy subject. Hence, in the resulting disconnectome map, the value in each voxel takes into account the interindividual variability of tract reconstructions in controls, and it indicates a probability of disconnection from 0 to 100% for a given lesion. We applied a threshold above 50% chance level to obtain the final disconnection map for each patient. We then used the statistical non-parametric mapping (SnPM, http://warwick.ac.uk/snpm), a SPM toolbox which performs nonparametric statistics on neuroimaging data[31]. The present analysis used the SPM12 and SnPM13 versions of these applications. We run a separate analysis for each of the behavioral outcomes of interests (BLT, PPS and as well as AAT). Right and left brain lesions were analyzed separately. Disconnectome maps were considered as dependent variables within the general linear model implemented in SnPM, in order to test the difference between groups of impaired versus unimpaired patients in terms of disconnected brain regions. Demographic (age), clinical (lesion size, sensory and motor deficits) and neuropsychological (neglect) data were considered in the model as control covariates. Results that survived 5000 permutations testing were controlled at cluster level for family-wise error rate ($p>0.95$). The involvement of the anatomical structures emerging as significant from the regression analysis results were mapped onto tractography reconstructions of white matter pathways[28]. We used Tractotron to quantify the severity of the disconnection by measuring the probability of the tract to be disconnected[29]. We applied a threshold above 50% chance level and a proportionof overlap between the lesion's volume and the tract's volume > 5%. Disconnection maps were segmented, and grey matter disconnected regions were identified using the Anatomical Automatic Labeling in SPM. Only clusters > 30 contiguous voxels were considered to be clinically relevant.

2. SUPPLEMENTARY RESULTS

2.1 Peripersonal Space: audito-tactile interaction task.

Preliminary analyses on unimodal tactile perception. Two preliminary analyses on unimodal RTs were conducted, one

on accuracy and one on RTs to unimodal tactile stimuli in the two groups.

First, we calculated the percentage of trials correctly answered with respect to the total of the administered tactile trials, as a general index of accuracy. Based on the index of accuracy, we decide to exclude 4 patients because they had an accuracy level below 60%. Once excluded these subjects, the group average accuracy was 98.05% and 98.3% for the left and right limb in controls, respectively, and 88.5% and 92.3% for the affected and unaffected limb in patients, respectively. The model (R^2=0.75, F(3, 78)=12.54, p<0.001) showed a main accuracy difference between patients and controls (all p values <0.012), a significant difference between the affected and non-affected limbs in patients (p=0.007), and no difference between limbs in controls (p=0.99). These results are expected and in line with a reduced sensory sensitivity for the controlesional side due to the lesion (see clinical scores, Supplementary table 2).

Secondly, we compared RTs to unisensory tactile stimuli for each temporal delay with respect to sound onset in the two groups, by applying linear mixed model with "limb" (4 levels coded as nested: affected/unaffected for patients, left/right for controls) and "temporal delays" (corresponding to near, medium and far distance) as factors, and participants as random factor. The model (R^2= 0.82) on unimodal RTs revealed a significant effect of "limb" and "temporal delays" (corresponding to near, medium and far distance) ("limb": F (3, 167)=19.42, p<0.001; "temporal delays": F (2, 375)=5.61, p<0.01), without a significant interaction between the two factors (F (6, 375)=0.91, p=0.49). Post hoc comparisons revealed that RTs on the affected limb were significantly slower than those of the unaffected limb in patients (p<0.0001) and those of both limbs in controls (p<0.001, all other p values >0.08) (see Supplementary figure 1).

Moreover, a general reduction in RTs to the last temporal delay (corresponding to the closest position) with respect to those presented at the medium delay (p=0.0032; all other p values >0.08) was present independently of the group, probably related to an expectancy effect for unimodal tactile stimuli (i.e., faster RTs for late tactile targets[23,32]). The same results were obtained if considering separately RBD (R^2=0.80; "limb": F (3, 133)=18.14, p<0.001; " temporal delays": F (2, 265)=5.38, p<0.01) and LBD (R^2=0.92; " limb": F (3, 116)=13.97, p<0.001;"temporal delays": F (2, 115)=4.82, p<0.01), thus suggesting no effects of the lesioned hemisphere on unimodal tactile processing.

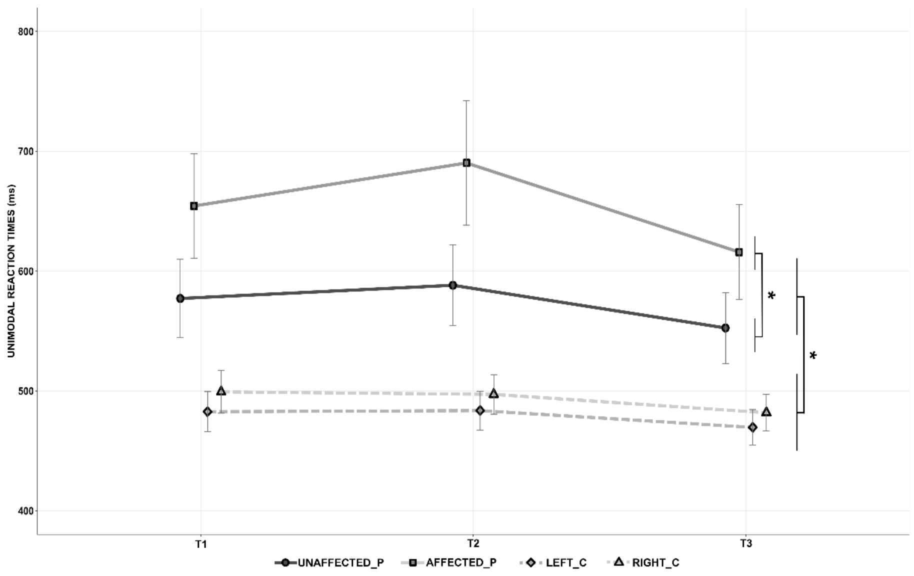

Supplementary figure 1. *Comparison on the unimodal tactile RT (mean ± standard errors, ms) as a function of the temporal delay (T1, T2, T3) in patients (UNAFFECTED/AFFECTED_P) and controls (LEFT/RIGHT_C) (Linear mixed models: F (3, 167)=19.42, p<0.001). RTs on the affected limb were significantly slower than those on the unaffected limb in patients (p<0.0001) and those on both limbs in controls (p<0.001).*

Multisensory PPS processing in all patients, comparison against the baseline. We tested if the corrected bimodal RTs at each of the three distances (NEAR, MEDIUM, FAR) were significantly lower than the baseline (i.e. the fastest reaction time for unimodal tactile stimuli taken as a baseline and indicated as zero, see Figure 2 in the main text or Supplementary figure 3), in patients and controls, by using one-sample t-test against the zero (one-tailed, lower than zero), Bonferroni corrected (alpha set at 0.05/6=0.008, 6 comparisons by considering the 3 distances for both sides in each group). In line with previous works (e.g.[12,15,18,33]), values below the baseline indicate a multisensory facilitation effect on tactile RTs due to auditory stimulation. Comparisons showed that corrected bimodal RTs in the controls are significantly lower than the baseline at the near and medium distances (all p values < 0.0004), but not at far distance (all p values>0.07), thus indicating that controls showed a multisensory facilitation in responding to bimodal RTs with respect to the baseline when the tactile stimuli were associated with sounds at medium (D2) and at near (D1) distances from the body. In contrast, in patients, corrected bimodal RTs were significantly lower than the baseline only in the near distance for the unaffected side (near: p=0.003; medium and far: all p values >0.08), but they were never significantly smaller than the baseline on the affected side (all p values >0.15), thus indicating no significant multisensory facilitation for the contralesional limb.

Multisensory PPS processing in LBD and RBD patients. Due to the prevalence of spatial disorders typically present in RBD, we explored the bimodal data by considering separately LBD (n=17) and RDB (n=22) patients (see the main text for the model considering all the patients together). The model comparing LBD and healthy controls (R2=0.65) show a main effect of distance (F(2, 82)=10.55, p<0.001, age: p=0.83). Post hoc comparisons suggest faster RTs when tactile stimuli were presented together with near or medium sounds, that is when auditory stimuli were closer to the body, rather

than when presented with far sounds (near versus far: p=0.0001; medium versus far: p<0.001). No significant difference was found between RTs obtained when sounds were delivered in medium and in near position (p=0.51). Crucially, no significant difference between the two groups emerged ("limb": F(3, 142)=1.29, p=0.028; "limb" X "distance": F(6, 190)=1.08, p=0.38), thus suggesting a similar PPS representation in LBD patients and controls.

In contrast, the model run on RBD patients and controls (R2=0.55) indicates a main effect of distance (F(2, 102)=12.63, p<0.001, age: p=0.64), but crucially also an effect of "limb" (F(3, 163)=7.70, p<0.001). The main effect of "distance" indicated that RTs were faster when tactile stimuli were presented associated with near or medium sounds, rather than with far sounds (near versus far: p<0.0001; medium versus far: p=0.001). Again, no significant difference emerged in RTs obtained with sounds in a medium and near distance (near versus medium: p=0.20). However, while no difference emerged in healthy controls between right and left hand (p=0.99), a difference emerged between the affected and the unaffected side in RBD patients (p=0.001), with slower RTs for the affected side, which were never facilitated with respect to the unimodal baseline responses (see Supplementary figure 2). In addition, RTs on the affected side in patients were significantly slower than those of both limbs in controls (all p values<0.01), while RTs on the unaffected side was comparable to controls (all p values<0.56). This suggests an impaired PPS representation for the affected limb in patients with RBD.

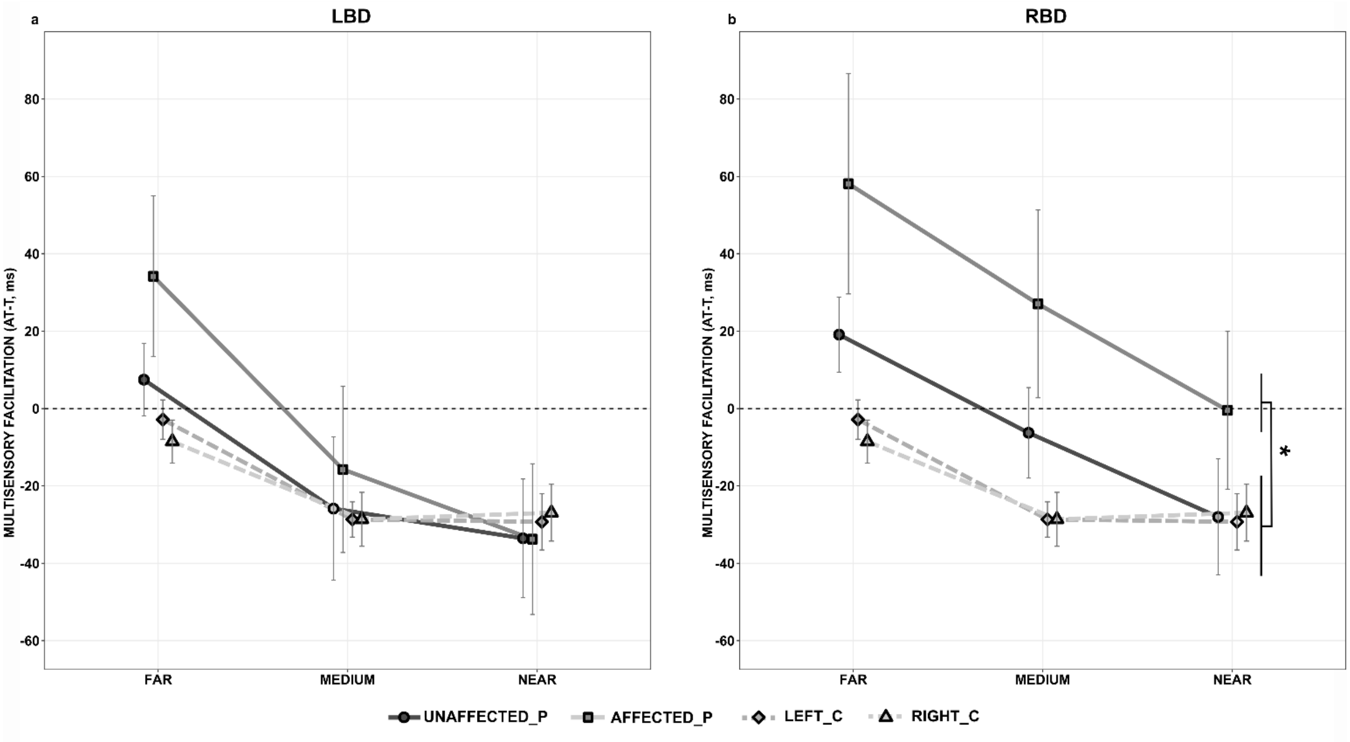

Supplementary figure 2. *Results of the explorative analysis at the PPS task in LBD and RDB (UNAFFECTED/AFFECTED_P) and controls (LEFT/RIGHT_C). Audio-tactile (AT) RTs (mean ± standard errors, ms) corrected for the unimodal tactile baseline (T, the fastest RTs to unimodal tactile stimulation, dashed line, 0, the baseline, AT-T) are shown as a function of the sound distance (FAR, MEDIUM and NEAR). Negative values indicate a multisensory facilitation effect in responding to audio-tactile stimuli with respect to the unimodal tactile baseline. The asterisk indicates a significant difference between the affected and the unaffected side in RBD patients (linear mixed model: F(3, 163)=7.70, p<0.001), with slower RTs for the affected side, independently of the temporal delays from the sound onset (p=0.001).*

Distortions in PPS representations in RBD with signs of neglect. Given the result of a significant PPS distortion in RBD

than in LBD patients (see above), we explored the possibility that this finding was linked to cognitive impairments typically occurring in RBD, such as visual hemispatial neglect. For this reason, we compared results obtained at the audio-tactile interaction task in RBD patients who presented or not visual hemispatial neglect. Concerning visual hemispatial neglect, we considered impaired a patient if he/she showed pathological scores at least in one of the clinical tests used to evaluate it, i.e. the Bell cancellation test (asymmetry score, see[34]) and at the visual field/neglect subtest of the Test of Everyday Attention (TEA, latency and accuracy scores, asymmetry score[8–10]). Baseline-corrected audio-tactile RTs were compared in RBD patients with (N+, n=6) or without (N-, n=15) signs of neglect by using LMM with distance, the presence of neglect (N+ and N-) and limbs (affected, unaffected) as fixed factors and patients as random factor. A triple interaction among distance, presence of neglect and limbs emerged (R^2=0.84; F(3, 84)=4.218, p=0.018). To explore this interaction, we run separate analyses on N+ and N- patients with distance and limbs as fixed factors and patients as random factors. While the expected effect of distance emerged in N- patients (R^2=0.83; F(2, 60)=10.139, p<0.001), with RTs to tactile stimuli associated with near sounds faster than those associated with medium (p=0.0156) and far sounds (p=0.0001; near versus medium: p=0.26), an interaction between distance and limbs emerged in N+ patients (R^2=0.88; F(2, 24)=4.110, p=0.029). In N+ patients no significant effect of sounds on tactile responses on the affected limb emerged, i.e. no significant differences on RTs associated with near, medium or far sounds (all p values >0.97), while the expected significant facilitation effect due to near sounds was observable on the unaffected limb, at least with respect to the far sounds (near versus far: p=0.0023; medium versus far: p=0.076; near versus medium: p=0.297).

2.2 Explicit body representation task: the avatar adjustment task (AAT)

60 stroke patients included for the supplementary task AAT (mean: 56.4 ± 13.9, range: 23-81) were compared with those collected in 47 age-matched controls (mean: 54.7 ± 22.6, range: 23-86, Mann-Whitney test: p=0.52).

Estimated dimension. The model run to explore a possible effect of the brain lesion in patients (fixed factors: body parts dimension, side: affected and unaffected, brain lesion: RBD/LDB) revealed a difference between LBD and RBD patients (R^2=0.91; F(3, 240)=7.97, p<0.001). Thus, in order to explore any difference between patients and the age-matched controls, we run separate analyses for LBD (n=26) and RBD (n=34) patients.

LBD. When LBD patients and controls were compared, a significant interaction between "body parts dimensions" and "limbs" (4 levels coded as nested: affected/unaffected for patients, left/right for controls) emerged (R2 =0.90, F (9, 208.60)=9.557, p<0.001). To explore this interaction, we ran separate analyses for each body part dimension with limbs as factor.

The *arm length* (R^2 =0.92, F (3, 81.78) = 16.25, p<0.001) was similarly perceived on both sides in patients (p=0.17), but it was always more underestimated with respect to controls (all p< 0.01). Differently from patients, left and right side diverge in controls (p<0.001), with a higher bias on the left side.

The *arm width* (R^2 =0.91, F (3, 80.29) = 8.92, p<0.001) of the affected side was perceived similar to unaffected side in

patients ($p=0.33$), and also to the right ($p=0.22$) and left limb ($p=0.59$) of controls. As for the arm length, while affected and unaffected side in patients were not significantly different, a left-right significant difference emerged in controls ($p<0.001$), with a higher bias on the left side.

Instead, a significant difference between the two sides emerged in patients for the *hand length* ($R^2=0.82$, $F(3, 94.75)=5.40$, $p=0.002$), with higher underestimation on the affected limb rather than the unaffected one ($p=0.002$) or the left limb in controls ($p=0.016$, right in controls versus affected: $p=0.23$). A left-right significant difference emerged in controls ($p=0.02$), with a higher bias on the right side.

No significant effects was found regarding the *hand width* ($R^2=0.75$, $F(3, 102.94)=0.80$, $p=0.49$; age: $p=0.70$) (Supplementary figure 3).

RBD. When RBD patients and controls were compared, a significant interaction between body part dimension and limbs emerges ($R^2=0.91$, $F(9, 223.92)=11.09$, $p<0.001$), so that separate analyses for each body part dimension with limbs as factor (4 levels coded as nested: affected/unaffected for patients, left/right for controls) were run.

For *arm length*, a significant difference between the affected and the unaffected limb emerged ($R^2=0.88$, $F(3, 94.74)=21.25$, $p<0.001$) in patients, with a higher underestimation on the affected side rather than on the unaffected one ($p<0.001$) and compared to both sides in controls (both p values<0.001). In controls, underestimation was larger in left rather than right limb ($p<0.001$).

Also for the *arm width* a significant effect emerged ($R^2=0.93$, $F(3, 86.34)=11.44$, $p<0.001$), but in this case the effect was driven by a limb difference in controls, with higher overestimation for the left rather than the right limb ($p<0.001$), while no significant differences emerged between RBD patients and controls (all p values >0.05).

Similar results were obtained for the *hand length* ($R^2=0.85$, $F(3, 97.14)=4.38$, $p=0.006$; age: $p=0.030$), with no limb difference in patients ($p=0.10$), but higher underestimation for the right rather than the limb side in controls ($p=0.013$). No significant effect was found regarding the *hand width* ($R^2=0.83$, $F(3, 99.27)=1.48$, $p=0.23$) (Supplementary figure 3).

In order to synthesize the data between the two upper limb parts and dimensions, we used a similar *normalized shape index* as for the BLT.

Normalized shape index. Separate analyses for LBD and RBD patients were performed due to a significant effect of the lesion lateralization ($F(1, 120)=13.18$, $p<0.001$).

LBD. A preliminary analysis considering together the hand and the arm in patients and controls showed an effect due to the body part ($F(3, 155.03)=16.35$, $p<0.001$), so that separate analyses for hand and arm were run.

A group effect emerged for the normalized shape index of the *arm* ($R^2=0.91$, $F(3, 82.45)=20.29$, $p<0.001$), with a larger, not lateralized (affected versus unaffected: $p=0.12$) bias in patients that was different from the right (both p values $p<0.001$), but not the left (p values $>.11$) limb of controls. This difference was due to the presence of a significant limb

difference in controls (p<0.001), who showed a stronger bias for the left, non-dominant hand (Supplementary figure 3).

In contrast, no significant effects emerged for the hand (R^2 =0.68, F (3, 108)=2.66, p=0.05).

RBD. A preliminary analysis considering together the hand and the arm showed an effect due to the body part (R^2 =0.85, F (3, 173.24)=17.17, p<0.001), so that separate analyses for hand and arm were run.

A significant effect emerged for the normalized shape index of the arm (R^2 =0.90, F (3, 91.35)=25.31, p<0.001),with a bias for the patients' affected limb that was larger as compared to the bias on the unaffected side (p=0.0014) and to that of both sides in controls (all p values < 0.008).

Considering the *hand*, a significant effect emerged too (R^2 =0.78, F (3, 105.5)=4.66, p=0.004), with a bias on the affected hand in patient that tends to be higher than that in controls (affected versus right: p=0.004; versus left: 0.09). No significant limb difference was present in patients (p=0.32), while a slight difference emerged in controls (higher bias on the right, p=0.047) (Supplementary figure 3).

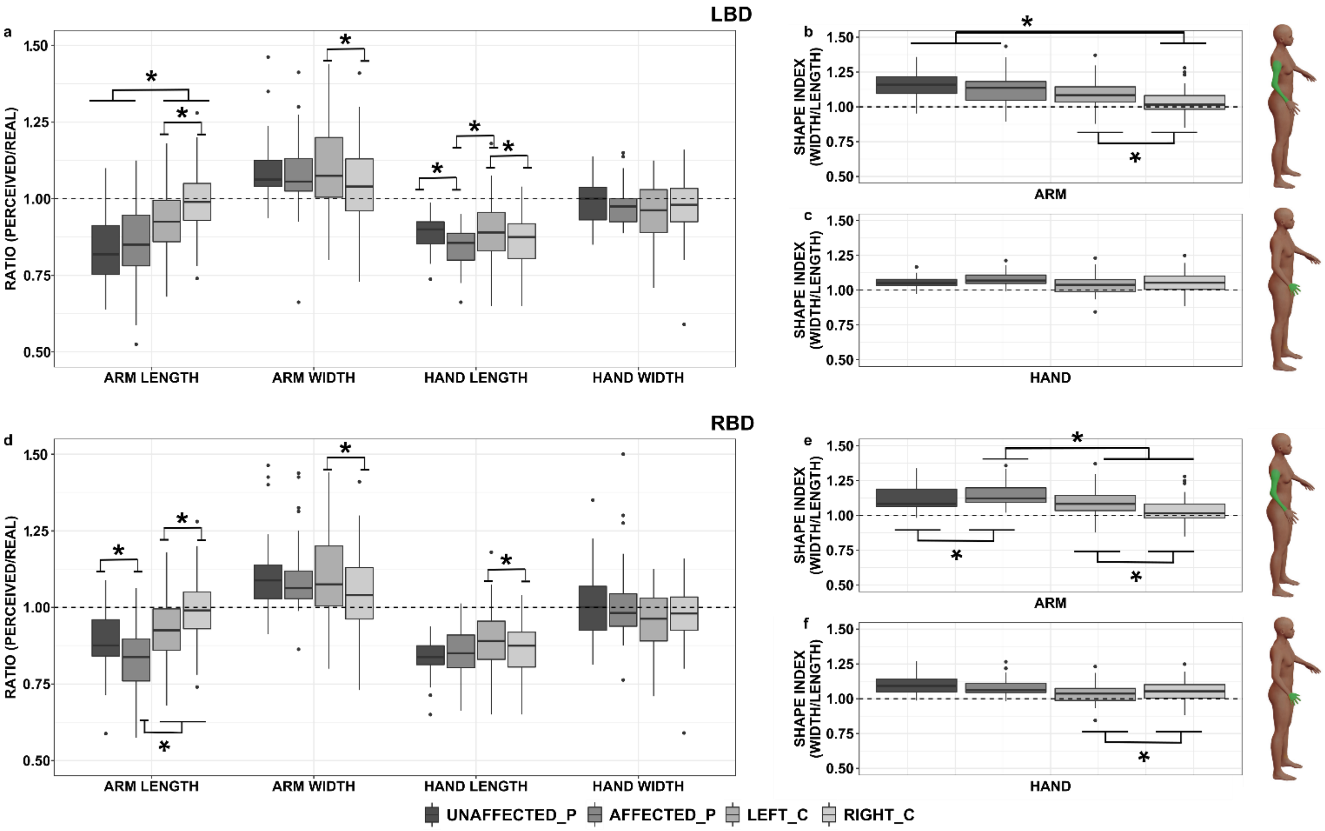

Supplementary figure 3. *AAT results on the estimated dimension (a-d) and the normalized shape index (b-c & e-f) for LBD (above, N=26) and RBD (below, N=34) patients (N=60, 21 females, UNAFFECTED/AFFECTED_P) and controls (N=47, 34 female, LEFT/RIGHT_C). The dashed line set at 1 indicates the correspondence between the perceived dimensions of one's own body and those of the observed avatar (an example is shown on the right). Asterisks above the boxes indicate the significant post hoc comparisons (all p values<0.02) within and between-groups (Linear mixed models: Estimated dimensions LBD: F (9, 208.60)=9.557, p<0.001; Shape Index LBD: F(3, 155.03)=16.35, p<0.001); Estimated dimensions RBD: F (9, 223.92)=11.09, p<0.001; Shape Index RBD: F (3, 173.24)=17.17, p<0.001). Data are represented with boxplots (internal line for median, whiskers for the largest (upper) and the smallest (lower) value ≥ 1.5 X IQR, black dots for outliers.*

To summarize, LBD and RBD patients showed a different pattern of distortions: LBD patients showed a similar perception bias for both the contralesional and the ipsilesional limb, while RBD patients showed a more severe bias for the controlesional limb than for the ipsilesional. However, a limb difference was found also in controls, indicating a higher bias for the non-dominant left limb than the for the dominant right limb. Together these findings suggest that an impairment affecting the non-dominant limb, as in RBD patients, exacerbates the asymmetry in perception present in healthy people at the AAT, while a lesion to the dominant arm, as in LBD, makes it as biased as the non-dominant one.

2.3 Prevalence, multiple regression and lesion analyses about the AAT.

Prevalence of alterations at the AAT. In order to provide indication about the distribution of deficits also at the AAT, we considered the estimated arm shape index (as for the BLT) as outcome of interest. The 25% of patients (15 out of 60 patients) were considered impaired relatively to controls (if patients' performance was 1.5 below the distribution of controls, see Method) at the AAT task. By considering the three tasks together, BLT , PPS and AAT, it emerges that the prevalence of distortions obtained by considering in at least one task was of about 48.3% (29 out of 60 patients, 54%, 21 out of 39 by considering only patients with data at the three tasks). Body representation deficits emerged as associated between tasks: 2 patients impaired at the BLT and AAT, and other 2 patients impaired at the AAT and the PPS. Dissociations were found as well, with 11 out of 15 patients selectively impaired only at the AAT.

M*ultiple linear regressions on the AAT*. The models were not significant for the AAT (p=0.13), thus suggesting that no significant predictors emerged for this outcome.

Lesion analysis on the AAT. Concerning the AAT index*,* the subtraction maps revealed no absolute difference (i.e. higher than 40%) in lesion prevalence between impaired and unimpaired patients. Similarly, the VLSM analyses and disconnection analysis did not reveal any association between distortions captured at the AAT and specific lesions (0 voxels survive threshold) or disconnection map.

2.4 Hierarchical clustering

From neuropsychological scores, the cluster analysis revealed no clear differentiation between the results at the different tests, with most scales significantly correlating among them without separate clusters emerging. The correlation matrix on neuropsychological tests (MMSE, Raven, FAB, go-no latency and accuracy) revealed a significant correlation (R 0.25 to 0.48 among MMSE, Raven and FAB and R 0.28 to 0.48 between FAB and all the other tests). Overall, the average R was 0.27, all correlations were positive and 6 out of 10 were statistically significant ($P<0.05$). This pattern of results suggests that the considered neuropsychological scores reflected a general measure of cognitive impairment. Thus, we considered an average score among all neuropsychological scales.

Hierarchical cluster analysis on motor scales (all the subscales for the MRC, MI, MAL, ARAT and FM, as well as the Box and Block Test subtest referring to the affected side) revealed two clusters with average within-cluster $R>0.6$. The first

cluster reflected mainly motor strength, by regrouping MRC and MI subtests, while the second cluster was related to the motor use, based on ARAT, MAL, FM, Box and Block scores. However, to ensure stability in multiple regressions, an average value between the two clusters capturing the general level of motor impairment was considered because of high (R>0.8) correlation (Pearson) between the clusters.

Hierarchical cluster analyses on sensory scores suggested two clusters, with average within-cluster R>0.5. The first cluster mainly reflected tactile acuity, by regrouping scores obtained at the two-points discrimination threshold for hand, arm and forearm. The second cluster was related to on proprioceptive ability (proprioceptive scores for the forearm, arm and hand at the Joint Position Sense) and tactile sensitivity (Bisiach and Fugl-Meyer subscale). The two indexes were obtained by averaging (normalized) values of scales within the clusters showed significant yet moderate collinearity (R<0.5) and were kept separated.

In summary, we considered a cluster of neuropsychological scores reflecting a general measure of executive functioning, a cluster capturing the global level of motor impairment and two clusters of sensory deficits concerning respectively tactile acuity and proprioception together with tactile sensibility.

2.5. Supplementary figures for results reported in the main text

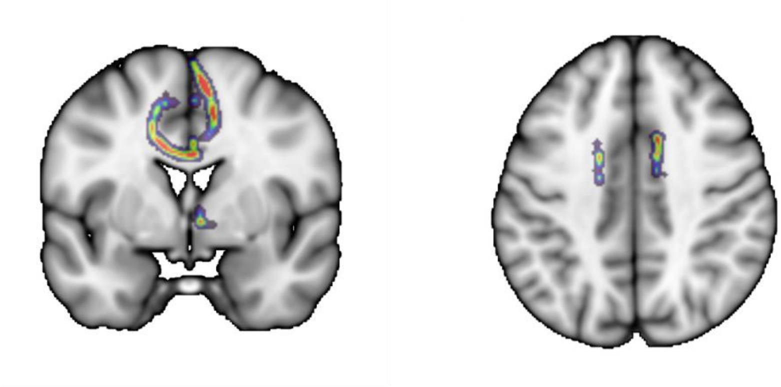

***Supplementary figure 4**. Disconnection maps for the BLT. Lesion of the corticospinal tact and of transcallosal fibers connecting the right supplementary motor area and the left anterior cingulate cortex were found to be associated with BLT deficits, if a more liberal threshold was considered (p < 0.01 uncorrected)*

2.6. Supplementary results in lesion analyses

In total, the clinical images were available for 38 patients. The sample includes both hemorrhagic (15) and ischemic (23) lesions, both on the right (19) and on the left (19) hemisphere (see Supplementary table 1). The time between the stroke andthe radiological exam varied, with images collected in acute (5 images, < 5 days), intermediate (13 images, < 3 months) and chronic (20 images, > 3 months) phase (phases reported in line with [35]).

<u>Voxel-based lesion-symptom mapping</u>. VLSM analyses did not reveal any behavioural outcomes associated with specific areas (0 voxels survive threshold). This null result might be due to the high variability in patients' images, combining

data from patients with available acute and chronic images[25,35]. Thus, the same analysis was repeated by considering only patients with images acquired in the acute and intermediate phase (n= 18, < 3 months from stroke). However, even when selecting only patients with acute images, the analysis did not reveal any significant cluster associated with our behavioral outcomes of interests.

Voxel-lesion symptom mapping on motor scores. When the VLSM including both acute and stroke images was performed on motor scores, i.e. MRC score (continuous values), significant results were obtained only if acute and intermediate images were included (z=-5.636700 +2.040213, 1136 voxels survived p <0.05 threshold). Results indicate significant clusters of voxel associated to motor deficits at the level of the anterior and posterior limb of the internal capsule.

3. SUPPLEMENTARY DISCUSSION

Distortions in the perceived metric characteristics of the affected upper limb captured by the AAT

Distortions in the perceived metric characteristics of the affected upper limb were captured not only at the implicit BLT but also at the more explicitly AAT. In the AAT participants provide explicit judgment of the perceived width and length of one's own upper limbs by instructing the experimenter to manipulate the corresponding body parts on an avatar. Similarly to the BLT, distortions at the AAT occur mainly at the level of the arm both in terms of underestimation of the length and distortions in the overall ratio between width and length. The similar bias concerning the arm at the BLT and the AAT is in line with previous data demonstrating in healthy participants that typical distortions in hand representation are captured by both an implicit task as the BLT, and also by more explicit metric task, as the "line length task" (e.g.[36]) . In this sense, the AAT and the "line length task" may share similarities because in both cases participants have to provide explicit judgments of their body parts dimension by modifying the visual characteristic of a model (avatar in our task, lines in the "line length task"[15]). However, in contrast with the results at the BLT, in the present work the AAT captured differences between LBD and RBD patients. LBD patients showed a similar perception bias for both the affected and the unaffected arm, while RBD patients showed a bias more severe for the affected limb rather than for the unaffected one. Interestingly, a limb difference was found also in controls, with higher bias in terms of length, width and overall ratio for the non-dominant left arm than for the dominant right arm. A higher bias for the left arm length in right-handed healthy participants mimics previous results captured by[37] with a task similar to the present AAT, in which participants estimated the length of their arm by explicitly instructing the experimenterto increase or decrease the length of a tape. According to the authors, this would reflect cortical hemispheric asymmetries observed in right-handed subjects, typically presenting greater cortical surface area in left sensory cortex and higher activation in left primary motor and sensory cortices for contralateral movements[37]. In this view, our results indicate that hand dominance could be at the origin of differences found between RBD and LBD patients in the AAT. Indeed, in our sample of right-handed participants, RBD patients present motor limitations at the (left) non-dominant limb, while LBD patients' deficits concern the (right) dominant limb. Thus, an impairment affecting the (left) non-dominant limb, as in RBD patients, exacerbates the asymmetric higher distortions in (left) non-dominant side observed in controls, thus leading to lateralized bias

specific for the (left) affected side. In contrast, a deficit to the dominant arm, as in LBD, makes it as biased as the non-dominant one,thus producing non-lateralized distortions, involving both arms. This lateralized bias in RBD is also in line with previous data showing a more asymmetric use of the two arms in right-handed stroke patients with RBD who use their unaffected (right) arm 4 times more frequently than the (left) affected ones, in contrast with a more symmetrical use of the two sides in LBD patients with matched degree of paresis (e.g.[38]). In agreement with this and given the visuo-somatosensory nature of the AAT, for which no upper limb movements were requested to perform the task, the influence of hand dominance is probably not directly linked to residual motor ability or dexterity per se, but it might mainly reflect use-dependent bodily related visual-somatosensory inputs, as visual-somatosensory feedback during motor experience. This could also explain the lack of correlations between AAT distortions and clinical scores about motor abilities and suggest that the possible involvement of functions crossing multiple domains (e.g. visual-somatosensory signals, arm use), not captured by clinical scores collected at the chronic phase of the disease.

4. SUPPLEMENTARY REFERENCES

1. Högg S, Holzgraefe M, Wingendorf I, Mehrholz J, Herrmann C, Obermann M. Upper limb strength training in subacute stroke patients: Study protocol of a randomised controlled trial. *Trials*. 2019;20(1). doi:10.1186/s13063-019-3261-3
2. Murphy MA, Resteghini C, Feys P, Lamers I. An overview of systematic reviews on upper extremity outcome measures after stroke. *BMC Neurol*. 2015;15(1). doi:10.1186/s12883-015-0292-6
3. Bisiach E, Vallar G, Perani D, Papagno C, Berti A. Unawareness of disease following lesions of the right hemisphere: Anosognosia for hemiplegia and anosognosia for hemianopia. *Neuropsychologia*. 1986;24(4):471-482. doi:10.1016/0028-3932(86)90092-8
4. Wolny T, Linek P, Michalski P. Inter-rater reliability of two-point discrimination in acute stroke patients. *NeuroRehabilitation*. 2017;41(1):127-134. doi:10.3233/NRE-171464
5. Goble DJ. Proprioceptive Acuity Assessment Via Joint Position Matching: From Basic Science to General Practice. *Phys Ther*. 2010;90(8):1176-1184. doi:10.2522/ptj.20090399
6. Spinnler H, Tognoni G. Standardizzazione e taratura italiana di test neuropsicologici. *Ital J Neurol Sci*. 1987;6(8):62-65.
7. Appollonio I, Leone M, Isella V, et al. The frontal assessment battery (FAB): Normative values in an Italian population sample. *Neurol Sci*. 2005;26(2):108-116. doi:10.1007/s10072-005-0443-4
8. Zimmermann P, Fimm B. Testbatterie zur Aufmerksamkeitsprüfung. *Würselen: Psytest*. 1992:1-1. doi:10.1007/978-3-642-54672-3_817-1
9. Zoccolotti P, Pizzamiglio P, Pittau PA, Galati G. Batteria di test per l'esame dell'attenzione. 1994.
10. Robertson IH, Ward T, Ridgeway V, Nimmo-Smith I. The structure of normal human attention: The Test of Everyday Attention. *J Int Neuropsychol Soc*. 1996;2(6):525-534. doi:10.1017/s1355617700001697
11. Fuentes CT, Longo MR, Haggard P. Body image distortions in healthy adults. *Acta Psychol (Amst)*. 2013;144(2):344-351. doi:10.1016/j.actpsy.2013.06.012
12. Serino A. *Peripersonal Space (PPS) as a Multisensory Interface between the Individual and the Environment, Defining the Space of the Self*. Vol 99. Elsevier Ltd; 2019:138-159. doi:10.1016/j.neubiorev.2019.01.016
13. Bassolino M, Serino A, Ubaldi S, Làdavas E. Everyday use of the computer mouse extends peripersonal space representation. *Neuropsychologia*. 2010;48(3):803-811. doi:10.1016/j.neuropsychologia.2009.11.009
14. Serino A, Bassolino M, Farnè A, Làdavas E. Extended Multisensory Space in Blind Cane Users. *Psychol Sci*. 2007;18(7):642-648. doi:10.1111/j.1467-9280.2007.01952.x
15. Sorrentino G, Franza M, Zuber C, Blanke O, Serino A, Bassolino M. How ageing shapes body and space representations: A comparison study between healthy young and older adults. *Cortex*. 2021;136:56-76. doi:10.1016/j.cortex.2020.11.021
16. Serino A, Noel JP, Galli G, et al. Body part-centered and full body-centered peripersonal space representations. *Sci Rep*. 2015;5. doi:10.1038/srep18603
17. Canzoneri E, Magosso E, Serino A. Dynamic Sounds Capture the Boundaries of Peripersonal Space Representation in Humans. *PLoS One*. 2012;7(9):3-10. doi:10.1371/journal.pone.0044306

18. Noel J-PP, Pfeiffer C, Blanke O, Serino A. Peripersonal space as the space of the bodily self. *Cognition*. 2015;144:49-57. doi:10.1016/j.cognition.2015.07.012

19. Förderreuther S, Sailer U, Straube A. Impaired self-perception of the hand in complex regional pain syndrome (CRPS). *Pain*. 2004;110(3):756-761. doi:10.1016/j.pain.2004.05.019

20. Galer BS, Jensen M. Neglect-like symptoms in complex regional pain syndrome: Results of a self-administered survey. *J Pain Symptom Manage*. 1999;18(3):213-217. doi:10.1016/S0885-3924(99)00076-7

21. Crema A, Bassolino M, Guanziroli E, et al. Neuromuscular electrical stimulation restores upper limb sensory-motor functions and body representations in chronic stroke survivors. *Med*. 2022;3(1):58-74.e10. doi:10.1016/j.medj.2021.12.001

22. Bastioni M, Re S, Misra S. Ideas and methods for modeling 3D human figures: The principal algorithms used by MakeHuman and their implementation in a new approach to parametric modeling. In: *COMPUTE 2008 - The 1st ACM Bangalore Annual Conference*. ; 2008. doi:10.1145/1341771.1341782

23. Sorrentino G, Franza M, Zuber C, Blanke O, Serino A, Bassolino M. How ageing shapes body and space representations: A comparison study between healthy young and older adults. *Cortex*. 2021;136:56-76. doi:10.1016/j.cortex.2020.11.021

24. Magni E, Binetti G, Bianchetti A, Rozzini R, Trabucchi M. Mini-mental state examination: A normative study in Italian elderly population. *Eur J Neurol*. 1996;3(3):198-202. doi:10.1111/j.1468-1331.1996.tb00423.x

25. de Haan B, Karnath H-O. A hitchhiker's guide to lesion-behaviour mapping. *Neuropsychologia*. 2018;115:5-16. doi:10.1016/J.NEUROPSYCHOLOGIA.2017.10.021

26. Suchan J, Rorden C, Karnath HO. Neglect severity after left and right brain damage. *Neuropsychologia*. 2012;50(6):1136-1141. doi:10.1016/j.neuropsychologia.2011.12.018

27. Foulon C, Cerliani L, Kinkingnéhun S, et al. Advanced lesion symptom mapping analyses and implementation as BCBtoolkit. 2018;7:1-17. doi:10.1093/gigascience/giy004

28. Rojkova K, Volle E, Urbanski M, Humbert F, Dell'Acqua F, Thiebaut de Schotten M. Atlasing the frontal lobe connections and their variability due to age and education: a spherical deconvolution tractography study. *Brain Struct Funct*. 2016;221(3):1751-1766. doi:10.1007/s00429-015-1001-3

29. Thiebaut De Schotten M, Tomaiuolo F, Aiello M, et al. Damage to white matter pathways in subacute and chronic spatial neglect: A group study and 2 single-case studies with complete virtual "in vivo" tractography dissection. *Cereb Cortex*. 2014;24(3):691-706. doi:10.1093/cercor/bhs351

30. Wang R, Benner T. *Diffusion Toolkit: A Software Package for Diffusion Imaging Data Processing and Tractography*.; 2007. http://cds.ismrm.org/ismrm-2007/files/03720.pdf. Accessed March 2, 2021.

31. Nichols TE, Holmes AP. Nonparametric permutation tests for functional neuroimaging: A primer with examples. *Hum Brain Mapp*. 2002;15(1):1-25. doi:10.1002/hbm.1058

32. Kandula M, Van der Stoep N, Hofman D, Dijkerman HC. On the contribution of overt tactile expectations to visuo-tactile interactions within the peripersonal space. *Exp Brain Res*. 2017;235(8):2511-2522. doi:10.1007/s00221-017-4965-9

33. Serino A, Noel JP, Galli G, et al. Body part-centered and full body-centered peripersonal space representations. *Sci Rep*. 2015;5(1):1-14. doi:10.1038/srep18603

34. Vallar G, Rusconi ML, Fontana S, Musicco M. Tre test di esplorazione visuo-spaziale: taratura su 212 soggetti normali. *Arch di Psicol Neurol e Psichiatr*. 1994;55(4):827-841.

35. Karnath HO, Rennig J. Investigating structure and function in the healthy human brain: validity of acute versus chronic lesion-symptom mapping. *Brain Struct Funct*. 2017;222(5):2059-2070. doi:10.1007/s00429-016-1325-7

36. Longo MR, Haggard P. Implicit body representations and the conscious body image. *ACTPSY*. 2012;141:164-168. doi:10.1016/j.actpsy.2012.07.015

37. Linkenauger S a., Witt JK, Bakdash JZ, Stefanucci JK, Proffitt DR. Asymmetrical body perception: A possible role for neural body representations. *Psychol Sci*. 2009;20(11):1373-1380. doi:10.1111/j.1467-9280.2009.02447.x

38. Rinehart JK, Singleton RD, Adair JC, Sadek JR, Haaland KY. Arm Use After Left or Right Hemiparesis Is Influenced by Hand Preference. 2009. doi:10.1161/STROKEAHA.108.528497
